# Reticular Imine‐Linked Coordination Polymers Based on Paddlewheel Diruthenium/Dirhodium Nodes: Synthesis and Metal‐Site Dependent Photocatalytic Reduction of CO_2_

**DOI:** 10.1002/cssc.202400885

**Published:** 2024-08-30

**Authors:** Chisa Itoh, Masaki Kitada, Mio Kondo, Shigeyuki Masaoka, Haruka Yoshino, Wataru Kosaka, Yusuke Ootani, Junko Matsuda, Momoji Kubo, Toyohiko J. Konno, Hitoshi Miyasaka

**Affiliations:** ^1^ Institute for Materials Research Tohoku University 2-1-1 Katahira, Aoba-ku Sendai 980-8577 Japan; ^2^ Department of Chemistry Graduate School of Science Tohoku University 6-3 Arama-ki-Aza-Aoba, Aoba-ku Sendai 980-8578 Japan; ^3^ Department of Chemistry School of Science Tokyo Institute of Technology NE-6, 2–12-1 Ookayama, Meguro-ku Tokyo 152-8550 Japan; ^4^ Division of Applied Chemistry Graduate School of Engineering Osaka University 2-1 Yamadaoka Suita Osaka 565-0871 Japan; ^5^ International Research Center for Hydrogen Energy Kyushu University 744 Motooka, Nishi-ku Fukuoka 819-0395 Japan

**Keywords:** Coordination Polymers, Ruthenium, Rhodium, CO_2_ Photoreduction, Catalysts

## Abstract

The paddlewheel‐type dimetal core ([M_2_]) is a ubiquitous motif in the nodes in coordination polymers (CPs) and metal‐organic frameworks (MOFs). However, their preparation has relied on ligand‐substitution‐labile metal ions owing to challenges associated with crystallization. Consequently, examples featuring ligand‐substitution‐inert metal ions, such as Ru or Rh, are scarce. This study presents the synthesis of novel reticular imine‐linked CPs incorporating the paddlewheel‐type diruthenium(II, II) ([Ru_2_^II,II^]; **1‐Ru**) or dirhodium(II, II) ([Rh_2_^II,II^]; **1‐Rh**) subunits. The synthetic approach involved a Schiff base dehydration condensation reaction between *p*‐formylbenzoate‐bridged [Ru_2_^II,II^] or [Rh_2_^II,II^] precursors (i. e., **CHO−Ru** and **CHO−Rh**, respectively) and 2,5‐dimethyl‐1,4‐phenylenediamine in a 1 : 2 ratio. The catalytic activities of **1‐Ru** and **1‐Rh** for the photochemical reduction of CO_2_ in a heterogeneous system depended on the metal site. The **1‐Ru** system exhibited exceptional selectivity, generating 3.0×10^4^ μmol g^−1^ of CO after 24 h of irradiation, whereas the **1‐Rh** system generated a lower amount of CO (3.2×10^3^ μmol g^−1^). The catalytic activity of **1‐Ru** ranked with that of all relevant catalytic systems. This study paves the way for the exploration of [Ru_2_^II,II^]‐ or [Rh_2_^II,II^]‐based polymers with open metal site‐dependent functional properties.

## Graphical abstract

Incorporating substitution‐reaction‐inert metal ions into coordination polymers (CPs) is challenging despite their potential for catalysts. This study reports the synthesis of novel reticular imine‐linked CPs via the Schiff base condensation of paddlewheel [M_2_^II,II^] (M=Ru, Rh) precursors with a phenylenediamine. Only [Ru_2_]‐based CP exhibits a high catalytic activity for the selective generation of CO in the photochemical reduction of CO_2_.

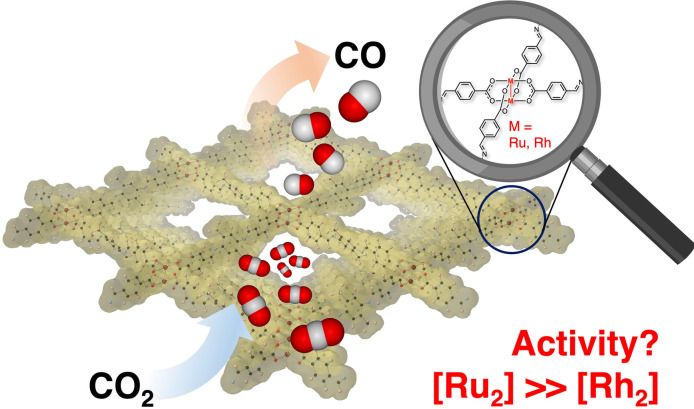

## Introduction

Molecular framework systems, denoted as coordination polymers (CPs)^[1]^ or metal‐organic frameworks (MOFs),^[2]^ have garnered substantial attention in the fields of chemistry and materials science. In these systems, the self‐assembly of metal ions and bridging ligands facilitate the homogeneous distribution of metal ions, resulting in well‐ordered crystalline structures with uniform geometries. Ligand‐substitution‐labile metal ions (hereafter referred to as labile metal ions) are suitable for constructing crystalline CPs/MOFs because a thermodynamically stable assembly can be formed without necessitating a high activation energy. The assembly reaction under rigorous conditions during hydrothermal synthesis is also available in the case with labile metal ions.[^3^, ^4^] Unlike labile metal ions, constructing homogeneous crystalline CPs/MOFs via instant ligand substitution using ligand‐substitution‐inert metal ions (hereafter referred to as inert metal ions) is challenging owing to the extremely high activation energies required for the ligand substitution processes. Moreover, these processes often entail multistage subprocesses, resulting in incompletely substituted forms, which may produce inhomogeneous materials or mixtures with defects. Consequently, a stepwise synthesis approach is a general strategy to mitigate these challenges. This approach involves the prior synthesis of metalloligands using inert metals, followed by the construction of frameworks via self‐assembly with other labile metal ions or subunits,[^5^, ^6^, ^7^, ^8^] thereby imposing restrictions on the synthetic route. Because the construction of cluster‐based CPs/MOFs is sophisticated, recent studies have reported reticular materials constructed via the combination of metal‐encapsulated rigid subunits featuring covalent bonding linkages, as observed in a specific type of covalent organic framework (COF).[^9^, ^10^, ^11^, ^12^, ^13^, ^14^, ^15^, ^16^] These materials, often denoted as metal‐covalent organic frameworks (MCOFs),^[17]^ combine the advantages of both CPs/MOFs and COFs; these benefits include providing inert metal ions/sites, structural rigidity, material stability, and structural porosity, resulting in synergistic properties. Additionally, the potential applications of MCOFs are being explored by harnessing their characteristics, such as covalent conjugation and open metal sites.[^18^, ^19^, ^20^, ^21^, ^22^, ^23^, ^24^, ^25^]

**Figure 1:**
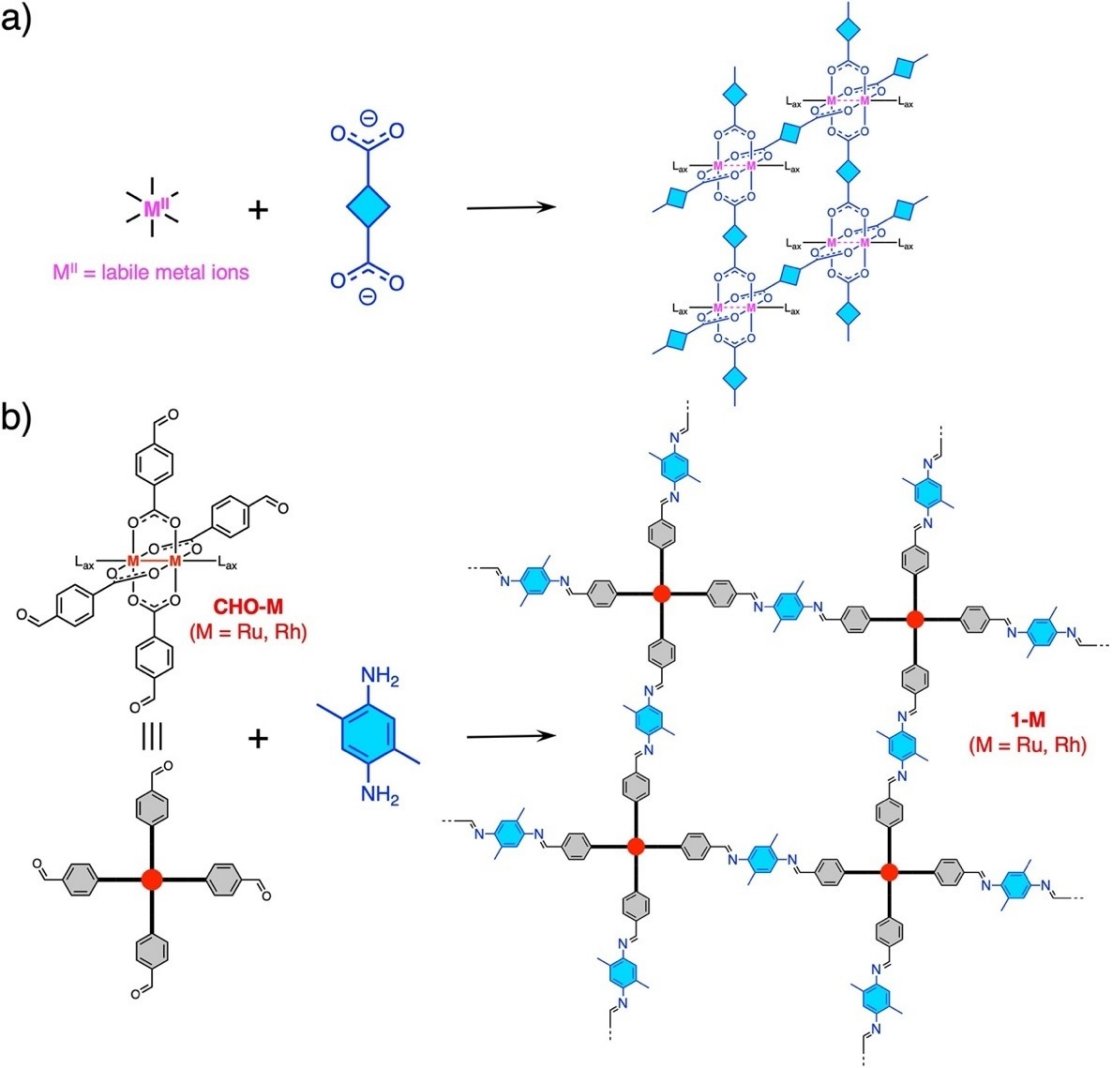
Schematic of synthetic routes for metal‐organic frameworks (MOFs) with paddlewheel‐type [M_2_] nodes. a) Typical MOF assembly formed by labile metal ions and carboxylate bridging ligands. b) Synthesis of metal‐covalent organic frameworks (MCOFs, **1‐M**, M=Ru or Rh) via Schiff base condensation between **CHO−M** and 2,5‐dimethyl‐1,4‐phenylenediamine (Me_2_PDA).

A reticular network featuring a carboxylate‐bridged paddlewheel‐type dimetal node ([M_2_]) represents a typical CP/MOF skeleton; some reticular networks exhibit open metal sites at apical positions on the node. However, most of these frameworks incorporate labile metal ions, such as Cu^II^ and Zn^II^, because of their construction during the complexation process of CPs/MOFs (Figure 1a).[^26^, ^27^, ^28^, ^29^, ^30^] CPs/MOFs characterized by [Ru_2_] or [Rh_2_] cluster centers are exceptionally rare owing to the inertness of the metal centers.^[31]^ Exceptions include CPs that incorporate metalloligands containing [Rh_2_] subunit,[^32^, ^33^] self‐assembled layers featuring [Mo_2_],^[34]^ pillared layer frameworks built on [Ru_2_],^[35]^ and the well‐known three‐dimensional HKUST‐1 forms incorporating 1,3,5‐benzenetricarboxylate.[^36^, ^37^, ^38^, ^39^] Moreover, the catalytic functionality of paddlewheel‐type diruthenium(II, II) ([Ru_2_^II,II^])[^35^, ^40^, ^41^, ^42^, ^43^, ^44^] or dirhodium(II, II) ([Rh_2_^II,II^])[^32^, ^45^, ^46^, ^47^, ^48^, ^49^, ^50^, ^51^, ^52^, ^53^, ^54^] complexes has recently attracted attention, thereby highlighting their potential in CPs/MOFs for designing heterogeneous catalysts. Notably, studies on CO_2_ reduction using the [Ru_2_^II,II^]‐based catalysts have not been reported.

Because of the stability of the inert [M_2_] core, it functions as a metal‐encapsulated rigid subunit in MCOF. This stability can enable post‐synthetic modification via covalent bonding, opening new possibilities for MCOF design based on this inert [M_2_] subunit. Herein, we report a rational route for synthesizing reticular imine‐linked CPs featuring nodes comprised of paddlewheel‐type [Ru_2_^II,II^] (**1‐Ru**) or [Rh_2_^II,II^] (**1‐Rh**) subunits, which were constructed via the Schiff base dehydration condensation of [M_2_^II,II^{*p*‐(CHO)ArCO_2_}_4_(THF)_2_] (M=Ru, **CHO−Ru** or Rh, **CHO−Rh**; *p*‐(CHO)ArCO_2_^−^=*p*‐formylbenzoate; THF=tetrahydrofuran)^[55]^ and 2,5‐dimethyl‐1,4‐phenylenediamine (Me_2_PDA) in a 1 : 2 ratio (Figure 1b). The catalytic activities of **1‐Ru** and **1‐Rh**, functioning as heterogenous catalysts, for the photochemical reduction of CO_2_ were studied in a system comprising a photosensitizer ([Ir^III^(ppy)_3_]; ppy=2‐phenylpyridinate), proton source (2,2,2‐trifluoroethanol (TFE)), and sacrificial electron donor (1,3‐dimethyl‐2‐phenyl‐2,3‐dihydro‐1*H*‐benzo[*d*]‐imidazole (BIH)) in a CO_2_‐saturated solution of *N*‐methyl pyrrolidone (NMP). The results revealed that the catalytic activity was dependent on the metal site [M_2_]; the **1‐Ru** system selectively generated 3.0×10^4^ μmol g^−1^ of CO after 24 h of irradiation, while the **1‐Rh** system generated a low amount of CO (3.2×10^3^ μmol g^−1^). Notably, the catalytic activity and selectivity of **1‐Ru** ranked with the top class in other relevant MCOF‐based catalytic systems.

## Results and Discussion

**Figure 2:**
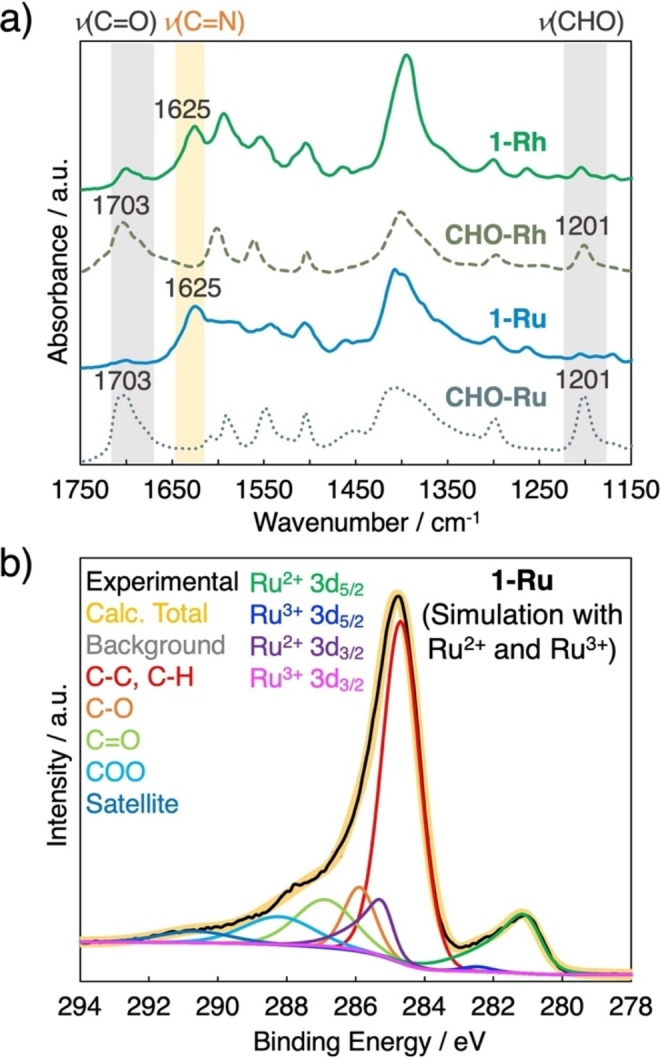
(a) Infrared spectra of **1‐M** and **CHO−M** (M=Ru or Rh). (b) X‐ray photoelectron spectra of **1‐Ru**. The simulated curves represent the C 1s, Ru^2+^ 3d, and Ru^3+^ 3d signals.

The **CHO−Rh** precursor was newly synthesized and characterized in this study (Tables S1 and S2), while **CHO−Ru** has been reported previously.^[55]^ **CHO−Rh** (+2THF as crystallization solvents for its single‐crystal crystallographic data)^[56]^ represents a typical paddlewheel [Rh_2_^II,II^] complex featuring THF as the apical ligands[^57^, ^58^, ^59^, ^60^] and was isostructural with **CHO−Ru** ⋅ 2THF,^[55]^ which crystallized in the triclinic *P*‐1 space group with an inversion center at the midpoint of the Rh−Rh bond (*Z*=1) (Figure S1). The Rh−Rh bond distance was 2.3856(6) Å, and the average Rh−O_eq_ (O_eq_=oxygen atoms of the carboxylate ligand) bond distance was 2.0348 Å, which is consistent with that of the [Rh_2_^II,II^] species.[^59^, ^60^] The infrared (IR) spectra exhibited intense characteristic peaks at 1703 and 1201 cm^−1^ corresponding to the vibrational mode of C=O (aldehyde), which is analogous to that observed for **CHO−Ru** (Figure 2a).

**Figure 3:**
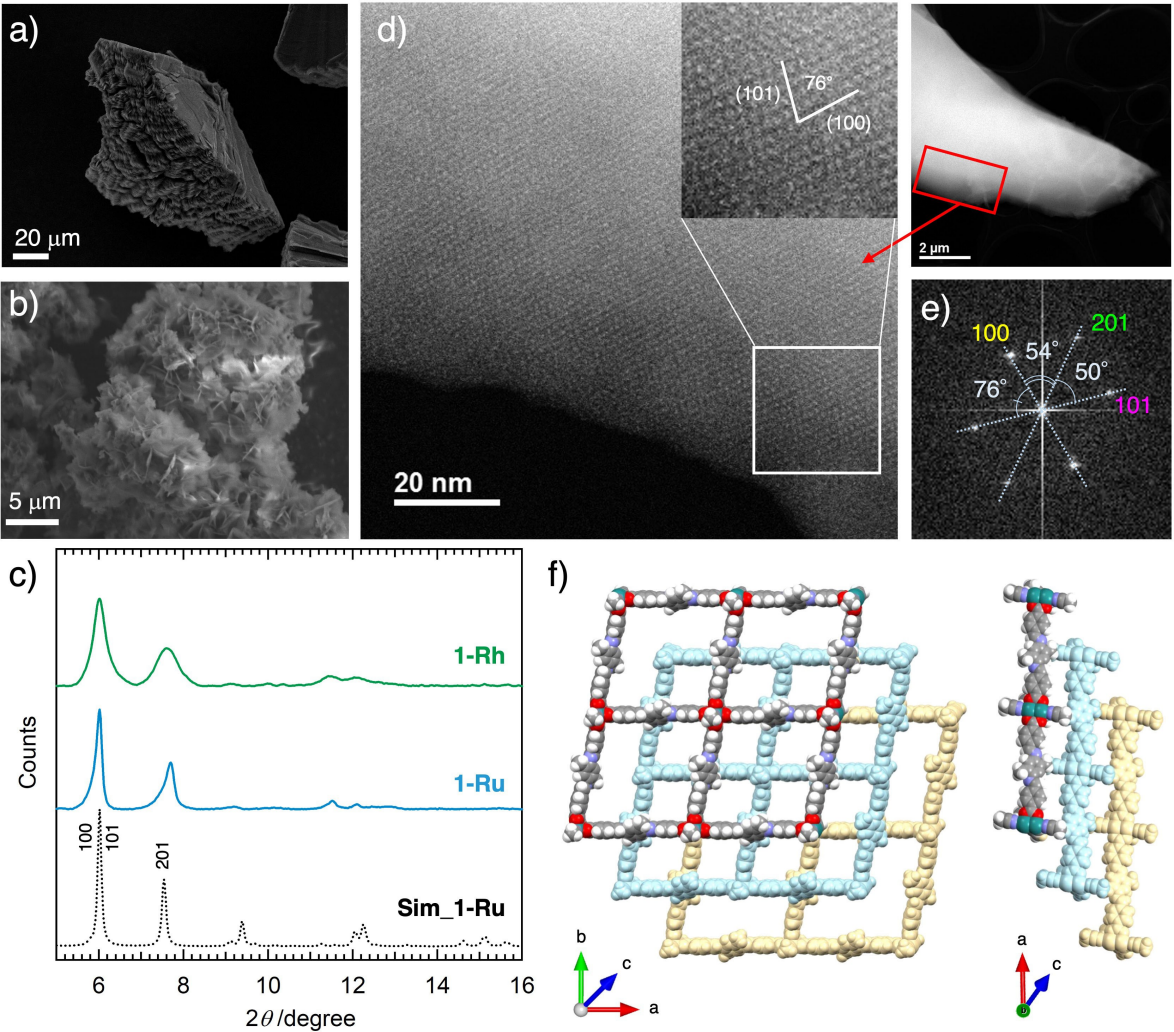
(a and b) Scanning electron microscopy (SEM) images of the as‐synthesized 1‐Ru and 1‐Rh samples, respectively (force constant: 16 N/m). (c) Powder X‐ray diffraction (PXRD) patterns of **1‐Ru** and **1‐Rh**. The simulated pattern for **1‐Ru** is overlaid, which is derived from the model structure shown in Figure 3f. (d) Scanning transmission electron microscopy (STEM) image of **1‐Ru**. (e) Selected area electron diffraction (SAED) pattern of **1‐Ru**. The labeled diffraction spots correspond to the *d*‐spacings of 1.43 (100), 1.25 (101), and 1.11 nm (201). (f) Model structure of **1‐Ru** (L_ax_=MeCN) constructed based on the combined data from the PXRD pattern, STEM image, and SAED pattern. The cell parameters are: *a*=23.88 Å, *b*=23.78 Å, *c*=17.96 Å, *α*=*β*=56.94°, and *γ*=100.30°.

The CPs **1‐Ru** and **1‐Rh** were synthesized by mixing a solution of **CHO−Ru** or **CHO−Rh** in acetonitrile (MeCN) and Me_2_PDA in dichloromethane (DCM) in a 1 : 2 ratio at room temperature. **1‐Ru** was produced using a slow diffusion technique in a narrow‐diameter glass tube, whereas **1‐Rh** was obtained by stirring the mixture at room temperature for several days (see Experimental Section). The crop‐shaped microcrystals of **1‐Ru** were produced in the central region of the glass tube, whereas **1‐Rh** was obtained as a precipitate. The scanning electron microscopy (SEM) images of **1‐Ru** and **1‐Rh** revealed that they crystallized homogenously with a microcrystalline morphology; however, the grains of **1‐Rh** were finer than those of **1‐Ru** (Figures 3a and S2 and 3b and S3 for **1‐Ru** and **1‐Rh**, respectively). Notably, heat treatment during the synthesis yielded precipitates immediately; however, the crystallinity of these precipitates was unsatisfactory because of their amorphous nature, unlike the compounds synthesized at room temperature.

The network structures of **1‐Ru** and **1‐Rh** were examined using various analytical methods. Wavelength‐dispersive X‐ray spectrometry (WDX) confirmed the distribution of N, C, O, and Ru/Rh atoms within the compounds (Figures S2 and S3). The IR spectra of **1‐Ru** and **1‐Rh** revealed the formation of imine bonds,^[55]^ which was confirmed by the characteristic vibrational peak at 1625 cm^−1^ for both compounds (Figure 2a). Notably, the peak corresponding to the vibrational mode of C=O and CHO (aldehyde), typically observed in **CHO−M**, was absent in the IR spectra of **1‐Ru**, while it was weakly observed in the IR spectra of **1‐Rh** (Figure 2). Nevertheless, neither compound exhibited distinct peaks in the 3200–3300 cm^−1^ range, which is characteristic of the N−H (amine) stretching mode in Me_2_PDA (Figure S4).

X‐ray photoelectron spectroscopy (XPS) analysis corroborated the divalent oxidation states of the Ru and Rh metal centers in **1‐Ru** and **1‐Rh**, respectively. Curve fitting simulations with potential electron exiting energy profiles confirmed the absence of other valent metal centers, such as Ru^3+^ species in **1‐Ru** (Figures 2b and S6c), which aligns with the characterization of the precursors **CHO−Ru** and **CHO−Rh** (Figures S5 and S6) as [Ru_2_^II,II^] and [Rh_2_^II,II^] complexes, respectively, using complementary techniques. The UV‐Vis spectra of **1‐Ru** and **1‐Rh** were obtained in solid‐state form, revealing the presence of isolated [Ru_2_] and [Rh_2_] units without any evidence of electronic conjugation or long‐range interaction (Figure S7). These findings indicate that **1‐Ru** and **1‐Rh** are isostructural. However, the condensation process in **1‐Rh** was shorter than in **1‐Ru**, resulting in shorter structural correlation lengths, smaller structural domains, and more unreacted defects within the network in **1‐Rh**. Nevertheless, the IR and XPS data demonstrated the successful formation and extensive distribution of imine‐linked networks across the long‐range lattices in both **1‐Ru** and **1‐Rh**, which were achieved remarkably under mild ambient temperature and pressure conditions. Powder X‐ray diffraction (PXRD) analysis was employed to validate the crystal structures of **1‐Ru** and **1‐Rh**. Measurements were performed on the as‐synthesized **1‐Ru** and **1‐Rh** (Figure 3c) and their solvent‐free counterparts (Figure S8) at room temperature. The presence of several low‐angle 2*θ* reflection peaks for the as‐synthesized compounds (Figure 3c) signifies their high crystallinity with well‐defined and oriented molecular lattice packing. The PXRD patterns revealed that **1‐Ru** and **1‐Rh** exhibited isostructural frameworks. Furthermore, the PXRD patterns of the solvent‐free compounds approximately resemble those of the as‐synthesized compounds (Figure S8), indicating the successful removal of interstitial solvents from the as‐synthesized compounds, as determined from the thermogravimetry (TG) measurements (Figure S9). The scanning transmission electron microscopy (STEM) images of **1‐Ru** reveal a lattice fringe with a *d*‐spacing of 1.31 nm (Figure 3d). Additionally, the selected area electron diffraction (SAED) pattern reveals diffraction spots corresponding to the *d*‐spacings of 1.43, 1.25, and 1.11 nm (Figure 3e).

Plausible reticular models were constructed based on the core structures of **CHO−Ru** and a reference structure of [Ru_2_(*p*‐MeArCO_2_)_4_(MeCN)_2_] (*p*‐MeArCO_2_^−^=*p*‐toluate; CH_3_CN=acetonitrile; Figure S10a) that were previously reported,^[61]^ and they were eventually optimized to accommodate the bridging phenyldiimine moiety. The axial coordination site was modeled to be occupied by MeCN due to the fact that the condensation reaction was carried out in a solution media containing MeCN (as shown in Figure S10). The layer stacking models, with variable stacking orientation along the *c*‐axis, were refined by the Le Bail profiles to achieve optimal agreement with the PXRD patterns. The lattice parameters were *a*=23.88 Å, *b*=23.78 Å, *c*=17.96 Å, *α*=*β*=56.94°, and *γ*=100.30° (Figure 3f; see the description on Model simulation). The characteristic diffraction peak at 2*θ*=6.02° was attributed to a combination of overlapping reflections from the (100) and (101) (*d*=1.47 nm) planes, which aligned well with the observed SAED pattern. The characteristic diffraction peak at 2*θ*=7.54° was attributed to the (201) reflection plane with *d*=1.17 nm. This analysis indicates an ABC stacking sequence for the layers in the crystal structure, where the guest‐accessible void space was estimated to be 64.5 % using the Mercury software (CCDC. Version 2024. 1.0) (Figure 3f and S11).

**Figure 4:**
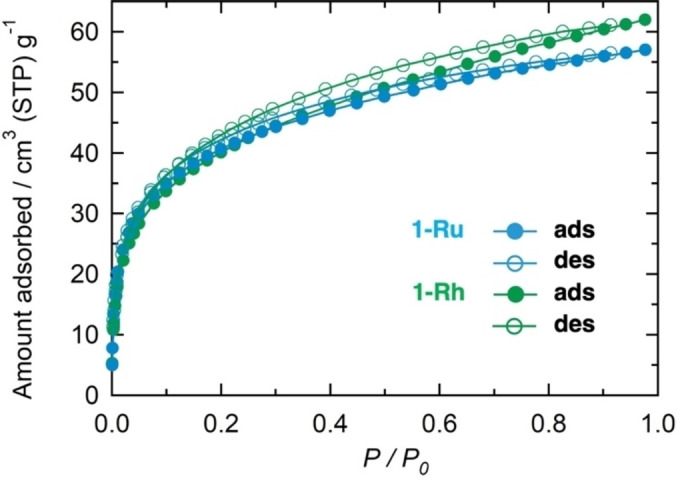
CO_2_ adsorption and desorption isotherms at 195 K for **1‐Ru** (blue) and **1‐Rh** (green).

The CO_2_ and N_2_ adsorption‐desorption isotherms for **1‐Ru** and **1‐Rh** were acquired at 195 and 77 K, respectively, following the *in situ* activation of the compounds at 403 K. Figures 4 and S12 show the CO_2_ and N_2_ isotherms, respectively. Both compounds exhibited remarkably similar isotherms; **1‐Ru** and **1‐Rh** exhibited CO_2_ adsorption capacities of 57 cm^3^ (STP) g^−1^ (2.5 mol mol^−1^) and 62 cm^3^ (STP) g^−1^ (2.8 mol mol^−1^), respectively, at *P*_CO2_=101.33 kPa. These characteristics indicate a typical Langmuir‐type adsorption profile (Figure 4). In contrast, the N_2_ adsorption was minimal (approximately 1.4 mol mol^−1^ at *P*_N2_=102.12 kPa; Figure S12). The small desorption hysteresis in the CO_2_ sorption profile can be attributed to the aggregation of the polycrystals. The Brunauer‐Emmett‐Teller analysis yielded surface areas of 160 m^2^ g^−1^ for **1‐Ru** and 150 m^2^ g^−1^ for **1‐Rh**. These findings indicate that both compounds possessed comparable porosity, although the pore sizes were not large.

**Figure 5:**
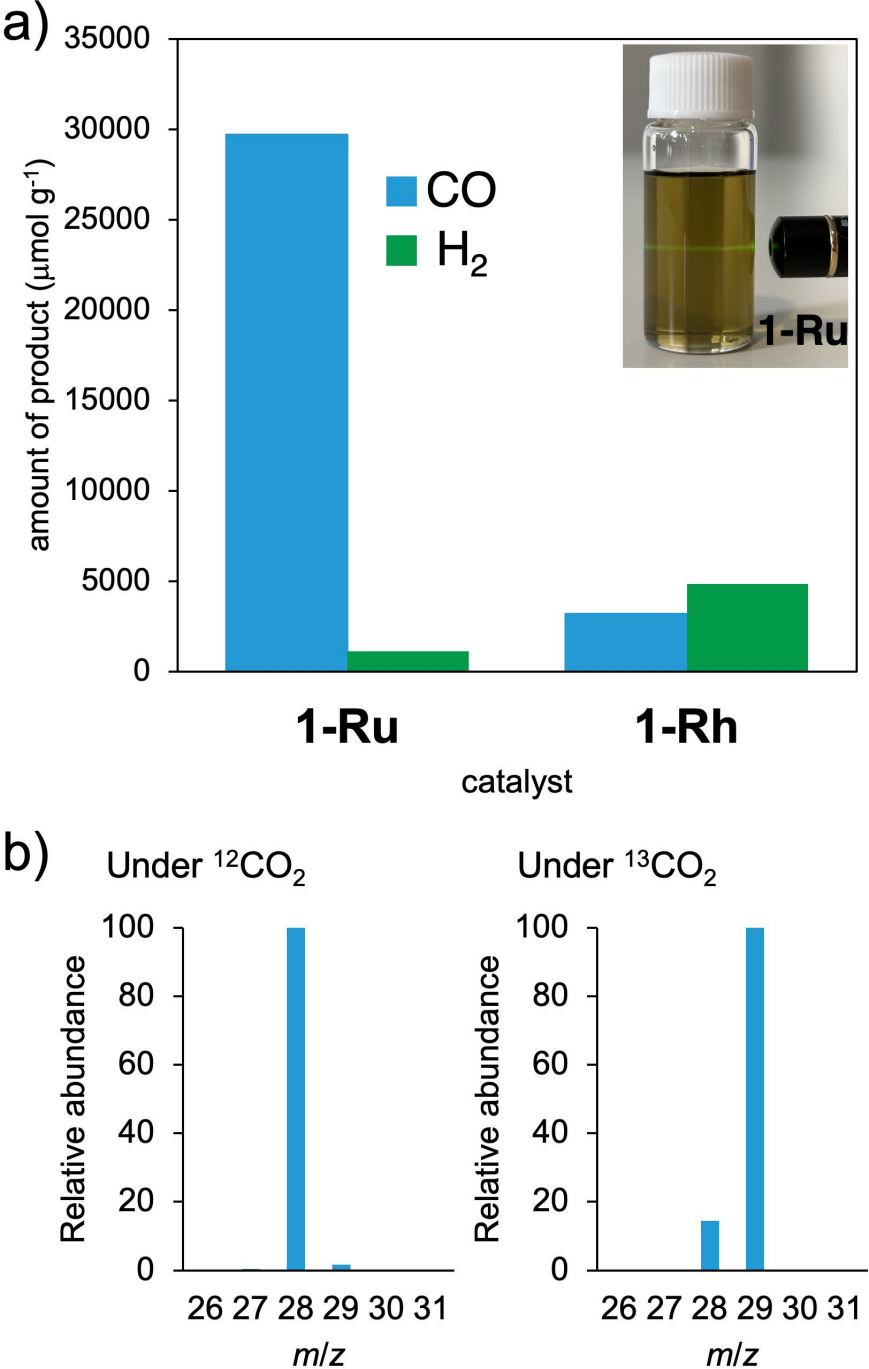
(a) Catalytic activities of **1‐Ru** or **1‐Rh** for the photochemical reduction of CO_2_ in an NMP solution containing [Ir(ppy)_3_], TFE, and BIH at 20 °C. The inset picture displays the Tyndall effect of dispersed **1‐Ru** in a NMP solution after sonicating for 8 hours. The solution was irradiated with a Xe lamp (400≤λ≤750 nm) under a CO_2_ atmosphere for 24 h. (b) Mass spectra of the CO gas generated in an NMP solution containing **1‐Ru**, [Ir(ppy)_3_], TFE, and BIH at 20 °C upon irradiation with a Xe lamp (400≤λ≤750 nm) under ^12^CO_2_ and ^13^CO_2_ atmospheres for 28 h.

We evaluated the photocatalytic activity of **1‐Ru** and **1‐Rh** for CO_2_ reduction under visible‐light irradiation (400 nm≤λ≤750 nm) (see Figure S13). We conducted the experiments under various conditions and with different component systems (see Tables S3–S5). Notably, one experiment condition produced an intriguing result that distinguishes between **1‐Ru** and **1‐Rh**. We performed the experiments in a heterogeneous system at 20 °C, where we dispersed **1‐Ru** or **1‐Rh** (0.025 mg) in an NMP solution (2 mL) containing [Ir(ppy)_3_] (0.1 mM) as the photosensitizer, TFE (0.1 M) as the proton source, and BIH (50 mM) as the sacrificial electron donor. We then analyzed the gaseous products of CO and H_2_ using gas chromatography. The **1‐Ru** system exhibited a distinct selectivity for CO and H_2_ products, with 1.2×10^4^ and 5.6×10^2^ μmol g^−1^ produced after 8 hours of visible‐light irradiation, respectively (Table S3). In comparison, the **1‐Rh** system produced 8.8×10^2^ and 4.1×10^3^ μmol g^−1^ of CO and H_2_, respectively (Table S4). After 24 hours of irradiation, the **1‐Ru** system yielded 3.0×10^4^ μmol g^−1^ of CO (production rate of 1.2×10^3^ μmol g^−1^ h^−1^) with trace amounts of H_2_ (1.1×10^3^ μmol g^−1^; production rate: 4.7×10 μmol g^−1^ h^−1^), while the **1‐Rh** system produced 3.2×10^3^ μmol g^−1^ of CO and 4.8×10^3^ μmol g^−1^ of H_2_ (Figure 5a and Table S5). The **1‐Ru** system demonstrated a selectivity for CO production of 96 %. This is, to our knowledge, the first report of CO_2_ reduction using a paddlewheel [Ru_2_^II,II^] species. The **1‐Rh** system, on the other hand, produced low amounts of CO and H_2_ in a 1 : 1.5 ratio (Figure 5a and Table S4). To confirm the origin of the CO, an isotopic labeling experiment was conducted using ^13^CO_2_ as the substrate in the **1‐Ru** system. Mass spectrum analysis revealed the formation of ^13^CO (Figure 5b), indicating that the detected CO originated from CO_2_ reduction.

Subsequently, several control experiments were carried out to understand the role of each component in the photocatalytic cycle. The results showed that in the absence of **1‐Ru**, [Ir(ppy)_3_], BIH, TFE, CO_2_, or visible‐light irradiation, minimal to no CO production was observed (Table S3, entries 3–8). These findings confirm that **1‐Ru** acted as the catalyst, [Ir(ppy)_3_] as a photosensitizer, BIH as a sacrificial electron donor, and CO_2_ as the substrate. Since many photocatalytic systems use a mixed solution of water, in which water molecules act as a proton source, the reaction was also tested in a mixed water solution. In this condition, CO was initially produced in the **1‐Ru** system (9.8×10^3^ and 1.1×10^3^ μmol g^−1^ for CO and H_2_, respectively, after 24 hours), although the yield was slightly lower compared to the condition with TFE (Table S5). Interestingly, the **1‐Rh** system produced more H_2_ in the presence of water (3.9×10^3^ and 4.6×10^4^ μmol g^−1^ for CO and H_2_, respectively, after 24 hours; Table S5), similar to some [Rh_2_]‐based systems.[^49^, ^51^, ^53^, ^54^, ^62^, ^63^, ^64^]

The stability and recyclability of materials **1‐Ru** and **1‐Rh** in the systems should be addressed. Although the solid substances remained stable for 24 hours, they ultimately proved to be subpar. We were unable to assess their quality in our systems due to the materials dispersing in solution as a colloid, as evidenced by the Tyndall effect (inset of Figure 5). The **1‐Ru** and **1‐Rh** compounds have a low‐dimensional layered structure, and it is believed that they were gradually exfoliated during the catalytic reactions.^[65]^ A residual powder obtained after filtering after one cycle reaction for 5 hours was analyzed using PXRD, but unfortunately, no peaks were detected, indicating being amorphous (Figure S14a). Despite this, the solid substrate was used in a second reaction, which showed similar catalytic activity after 5 hours (Figure S14b). It is worth noting that the FT‐IR data of powder samples of **1‐Ru** sonicated in a NMP solution for 8 hours were virtually identical to those of the pristine **1‐Ru** (Figure S14c). Thus, the two‐dimensional framework form is thought to be maintained even in a colloidal reaction solution.

Finally, we evaluated the catalytic efficiency of **1‐Ru** for the photochemical reduction of CO_2_ in comparison to other reported MCOF‐based photocatalytic systems.[^66^, ^67^, ^68^, ^69^, ^70^, ^71^, ^72^, ^73^] Our results, presented in Table S6, indicate that **1‐Ru** demonstrated superior catalytic activity when compared to the other reported systems.

## Conclusions

Reticular, layered CPs were constructed via Schiff base dehydration condensation of the **CHO−Ru** or **CHO−Rh** complex with Me_2_PDA, linking the metal centers at the nodes of the paddlewheel [Ru_2_^II,II^] or [Rh_2_^II,II^] cores, respectively (**1‐Ru** and **1‐Rh**, respectively). Despite the milder reaction conditions, the condensation process yielded significantly enlarged and well‐stacked layers in the isostructural frameworks of **1‐Ru** and **1‐Rh**. Comprehensive analyses and structural modeling of **1‐Ru** confirmed an ABC stacking arrangement of the layers, resulting in a porous structure that exhibited a CO_2_ adsorption capacity of approximately 3 mol mol^−1^. Furthermore, **1‐Ru** demonstrated exceptional catalytic activity for the photoreduction of CO_2_, selectively producing CO. This work opens new avenues for the development of functional materials based on the [Ru_2_] or [Rh_2_] centers within CP frameworks, highlighting the potential of [M_2_]‐based units for catalytic applications.

## Supporting Information

Structural characterization of **CHO−M** (M=Ru or Rh); additional SEM images and EMI for **1‐M**; IR, XPS, PXRD, and TG data for **1‐M**; structural model simulation for **1‐Ru**; N_2_ adsorption‐desorption isotherms for **1‐M**; image of custom‐designed photoreactor; and control experiments for the photochemical reduction of CO_2_ are shown in Figures S1–S14 and Tables S1–S6. Crystallographic data for **1‐Rh** (CCDC‐2342433) at 102 K

## Conflict of Interests

The authors declare no conflict of interest.

1

## Supporting information

As a service to our authors and readers, this journal provides supporting information supplied by the authors. Such materials are peer reviewed and may be re‐organized for online delivery, but are not copy‐edited or typeset. Technical support issues arising from supporting information (other than missing files) should be addressed to the authors.

Supporting Information

## Data Availability

The data that support the findings of this study are available in the supplementary material of this article.
