# Supplementary material for: Reticular Imine‐Linked Coordination Polymers Based on Paddlewheel Diruthenium/Dirhodium Nodes: Synthesis and Metal‐Site Dependent Photocatalytic Reduction of CO2
Source: ChemSusChem. 2024 Aug 30;17(23):e202400885. doi: 10.1002/cssc.202400885 (PMC11632573; doi:10.1002/cssc.202400885)
Supplement: Supplementary file 1 — Supporting Information [file CSSC-17-e202400885-s001.pdf]

ChemSusChem

Supporting Information

Reticular Imine-Linked Coordination Polymers Based on Paddlewheel Diruthenium/Dirhodium Nodes: Synthesis and Metal-Site Dependent Photocatalytic Reduction of CO_2

Chisa Itoh, Masaki Kitada, Mio Kondo, Shigeyuki Masaoka, Haruka Yoshino, Wataru Kosaka, Yusuke Ootani, Junko Matsuda, Momoji Kubo, Toyohiko J. Konno, and Hitoshi Miyasaka*

Supporting Information
©Wiley-VCH 2021
69451 Weinheim, Germany

Reticular Imine-Linked Coordination Polymers Based on Paddlewheel Diruthenium/Dirhodium Nodes: Syntheses and Photocatalytic Reduction of CO_2 Depending on the Metal Site

Chisa Itoh, Masaki Kitada, Mio Kondo, Shigeyuki Masaoka, Haruka Yoshino, Wataru Kosaka, Yusuke Ootani, Junko Matsuda, Momoji Kubo, Toyohiko J. Konno, and Hitoshi Miyasaka*

Abstract: The paddlewheel-type dimetal core ([M_2]) is a ubiquitous motif in the nodes in coordination polymers (CPs) and metal–organic frameworks (MOFs). However, their preparation has relied on ligand-substitution-labile metal ions owing to challenges associated with crystallization. Consequently, examples featuring ligand-substitution-inert metal ions, such as Ru or Rh, are scarce. This study presents the synthesis of novel reticular imine-linked CPs incorporating the paddlewheel-type diruthenium(II, II) ([$Ru_2^{II,II}$]; **1-Ru**) or dirhodium(II, II) ([$Rh_2^{II,II}$]; **1-Rh**) subunits. The synthetic approach involved a Schiff base dehydration condensation reaction between *p*-formylbenzoate-bridged [$Ru_2^{II,II}$] or [$Rh_2^{II,II}$] precursors (i.e., **CHO–Ru** and **CHO–Rh**, respectively) and 2,5-dimethyl-1,4-phenylenediamine in a 1:2 ratio. The catalytic activities of **1-Ru** and **1-Rh** for the photochemical reduction of CO_2 in a heterogeneous system depended on the metal site. The **1-Ru** system exhibited exceptional selectivity, generating 3.0×10^4 μmol g^{-1} of CO after 24 h of irradiation, whereas the **1-Rh** system generated a lower amount of CO (3.2×10^3 μmol g^{-1}). The catalytic activity of **1-Ru** ranked with that of all relevant catalytic systems. This study paves the way for the exploration of [$Ru_2^{II,II}$]- or [$Rh_2^{II,II}$]-based polymers with open metal site-dependent functional properties.

DOI: 10.1002/anie.2021XXXXX

Table of Contents

Experimental Section ········· S3
Table S1. Crystallographic data for **CHO-M** (M = Ru, Rh) ········· S5
Table S2. Relevant bond lengths on **CHO-M** (M = Ru, Rh) ········· S6
Figure S1. Structures of **CHO-Ru** and **CHO-Rh** ········· S7
Figure S2. Additional SEM images and elements mapping images for **1-Ru** ········· S8
Figure S3. Additional SEM images and elements mapping images for **1-Rh** ········· S9
Figure S4. IR in the range relevant to the N–H vibrational mode for **1-Ru** and **1-Rh** ·· S10
Figure S5. XPS for **CHO-Rh** and **1-Rh** ········· S11
Figure S6. XPS for **CHO-Ru** and **1-Ru** ········· S12
Figure S7. The solid-state UV-vis spectra of **1-Ru** and **1-Rh** ········· S13
Figure S8. PXRD patterns for solvent-free dried samples of **1-Ru** and **1-Rh** ········· S14
Figure S9. TGs for **1-Ru** and **1-Rh** ········· S15
Figure S10. Structural model simulation for **1-Ru** ·········S16
Figure S11. The pore volume of the model structure of 1-Ru.········· S17
Figure S12. Adsorption isotherms on N_2 for **1-Ru** and **1-Rh** ········· S18
Figure S13. Image of custom-made photoreactor ········· S19
Table S3. Control experiments for photochemical CO_2 reduction for 8 h by **1-Ru** ···· S20
Table S4. Control experiments for photochemical CO_2 reduction for 8h by **1-Rh** ····· S21
Table S5. Photocatalytic CO_2 reduction for 24 h by **1-Ru** and **1-Rh** ········· S22
Table S6. Comparison of catalytic activity of **1-Ru** and MCOFs ········· S23
Figure S14. Investigation on recyclability of the **1-Ru** material ········· S24
References on SI ········· S25

Experimental Section

Materials and Procedures

Materials

Syntheses were conducted under an inert atmosphere using the standard Schlenk line technique and a commercial glove box. All chemicals were purchased from commercial sources and were of reagent grade. The solvents were distilled under an N_2 atmosphere using a common drying agent. The starting material [$Rh_2(CH_3CO_2)_4(MeOH)_2$], used as the precursor for **CHO–Rh,** was synthesized following the reported methods.[1,2] Compound **CHO–Ru** was prepared according to a previously reported method.[3]

Synthesis of [$Rh_2^{II,II}$(*p*-CHOArCO$_2$)$_4$(THF)$_2$] (CHO–Rh)

A diglyme solution (20 mL) containing [$Rh_2(CH_3CO_2)_4(MeOH)_2$] (506 mg, 1 mmol) and *p*-formylbenzoic acid (1.2 g, 8.0 mmol) was refluxed for 2 h. After the solvent was evaporated under vacuum, the green residue was washed with *n*-hexane a few times and dissolved in a minimum amount of THF. The green THF solution was filtered and layered with *n*-hexane in a Schlenk tube with a diameter of 3 cm to obtain green block-shaped crystals after one week. The obtained crystal corresponds to **CHO-Rh**·2THF, which was evaluated by single-crystal X-ray crystallography. The crystal sample was dried *in vacuo* to measure elemental analysis. Yield: 42%. Elemental analysis (%) of [$Rh_2^{II,II}$(*p*-CHOArCO$_2$)$_4$(THF)$_2$]·1.5(H$_2$O) (**CHO-Rh**·1.5(H$_2$O)); $C_{40}H_{39}O_{15.5}Rh_2$: C, 49.35, H, 4.04; found: C, 49.39, H, 4.12; IR (KBr): $\nu(CO_2)$ = 1601 and 1401 cm^{-1}, ν(CHO) =1703 and 1201 cm^{-1}.

Synthesis of 1-Ru

A solution of Me$_2$PDA (272 mg, 2 mmol) in DCM (100 mL) was separated into 50 parts and placed in narrow-diameter glass tubes (ϕ 8 mm) to form the bottom layer. Subsequently, a mixture of DCM and MeCN (1:1 v/v, 1 mL) was added as a buffer (middle layer) for slow diffusion. Finally, a solution (2 mL) of [$Ru_2^{II,II}$(*p*-CHOArCO$_2$)$_4$(THF)$_2$] (377 mg, 0.4 mmol) in MeCN (100 mL) was carefully layered on the middle layer in each tube. The glass tubes were left undisturbed for three weeks to obtain brown, ball-shaped microcrystals of **1-Ru**. Yield: 39%. IR (KBr): $\nu(CO_2)$ = 1594 and 1395 cm^{-1}, ν(C=N) = 1625 cm^{-1}.

Synthesis of 1-Rh

A solution of [Rh_2(*p*-CHOArCO$_2$)$_4$(THF)$_2$] (41 mg, 0.04 mmol) in MeCN (10 mL) was added to a solution of Me$_2$PDA (22 mg, 0.16 mmol) in DCM (10 mL) and stirred at room temperature. Brown powder samples were obtained after stirring for five days. Yield: 33%. IR (KBr): $\nu(CO_2)$ = 1588 and 1415 cm^{-1}, ν(C=N) = 1625 cm^{-1}.

Characterization

IR spectra were recorded using KBr disks on a JASCO FT/IR 4200 spectrometer. Magnetic susceptibility measurements were conducted using a SQUID magnetometer (Quantum Design, MPMS-XL) in the range of 1.8–300 K. Polycrystalline samples were analyzed after embedding in liquid paraffin. Diamagnetic corrections were applied using the Pascal constants.[4,5] Thermogravimetric analysis curves were recorded on a Shimadzu DTG-60H instrument under an N_2 atmosphere. Samples were heated in the 298–673 K range at a rate of 5 K min^{-1}. XPS patterns were acquired using an AXIS Ultra DLD system from Shimadzu–Kratos Analytical Ltd. with a focused monochromatic Al–Kα X-ray source (1486.6 eV). The binding energy was calibrated using the C(1s) peak at 284.8 eV. The X-ray power, spot size, and analyzer pass energy were set to 150 W (15 kV, 10 mA), 300 × 700 µm, and 40 eV, respectively. SEM images were obtained using JEOL JXA-8530F (field emission electron probe microanalysis (FE–EPMA) and trace element analysis) and JEOL JSM-IT200 instruments. The FE–EPMA analysis was conducted using an acceleration voltage of 5–15 kV, with a minimal beam diameter (focused) in both measurements. Elemental mapping was performed with a dwell time of 30 ms and an acceleration voltage of 15 kV. STEM images were obtained using a JEM-ARM200F instrument (JEOL Ltd., Tokyo, Japan) at an acceleration voltage of 200 kV. The as-synthesized crop-like microcrystals of **1-Ru** were dispersed in the mother liquid via ultrasonication for crystallization (DCM and MeCN mixtures) and used as samples for STEM.

Single-crystal X-ray crystallography for CHO–Rh·2THF

Single-crystal XRD data for freshly obtained THF-including **CHO–Rh** (**CHO-Rh**·2THF) were collected at 103 K using a charge-coupled device diffractometer (Rigaku Oxford diffraction, XtaLAB Synergy Custom, MM7-HyPix-600HE) with multilayer mirror monochromated Mo–Kα radiation (λ = 0.71075 Å). A single crystal was mounted onto a thin Kapton film using Nujol and cooled under a stream of N_2 gas. The structure was determined using direct methods (SHELXT, version 2018/2)[6] and expanded using Fourier transform techniques. Full-matrix least-squares refinements on F^2 were executed based on observed reflections involving variable parameters and converged with unweighted and weighted agreement factors of $R_1 = \Sigma ||F_o| - |F_c|| / \Sigma |F_o|$ ($I > 2.00\sigma(I)$) and $wR_2 = [\Sigma(w(F_o^2 - F_c^2)^2) / \Sigma w(F_o^2)^2]^{1/2}$ (all data). Nonhydrogen atoms were refined anisotropically, and the hydrogen atoms were refined using a riding model. The Sheldrick weighting scheme was applied in this study. The neutral atom scattering factors reported by Cromer and Waber were used.[7] Anomalous dispersion effects were included in F_c,[8] and the values of $\Delta f'$ and $\Delta f''$ were consistent with those reported by Creagh and McAuley.[9] The values of the mass attenuation coefficients relied on those reported by Creagh and Hubbell.[10] All calculations were performed using the OLEX2 crystallographic software package.[11] These data were deposited as a crystallographic information file (CIF) at the Cambridge Data Centre under Supplementary Publication No. CCDC-2342433 for **CHO–Rh**·2THF.

PXRD measurements
PXRD patterns were acquired using a RIGAKU Ultima IV diffractometer with Cu–$K\alpha$ radiation (λ = 1.5418 Å). The ground samples were sealed in a soda glass capillary tube with an inner diameter of 0.5 mm, and the PXRD pattern was acquired with a step size of 0.02°.

Model simulation
The construction of the **1-Ru** model was based on the crystal structure of [Ru_2(*p*-MeArCO_2)$_4$(CH_3CN)$_2$] (Me–[Ru_2], *p*-MeArCO_2^- = *p*-toluate, as shown in Figure S9a).[12] Me–[Ru_2] is the only reported crystal structure of an MeCN diadduct of [Ru_2], and since **1-Ru** was prepared using MeCN, it served as a suitable reference. Two Me_2PDA groups were attached to each of the two adjacent groups of the four bridging *p*-toluates in Me–[Ru_2] via imine bonds. The NH atoms were bonded to the remaining two *p*-toluates (Figure S10b). The molecular model was optimized using Gaussian09[13] with the B3LYP functional.[14] The SDD basis sets were used for Ru[15,16] and a 6–31g(d) basis set for the other atoms.[17-19] Although the expected spin multiplicity for this molecule is a triplet, a singlet state was assumed for computational simplicity. This assumption was deemed to have negligible effects because no structural optimization of the central [Ru_2] part was performed. A two-dimensional planar structure (Figure S10c) was constructed by converting the vectors connecting the terminal nitrogen atoms of the optimized molecule into translation vectors a and b (Figures S9b and d, respectively). This step determined the lattice constants *a*, *b*, and γ.
The following was done in the next step: aligning the two-dimensional planes at suitable distances (*c*) and angles (α and β). The X-ray diffraction (XRD) results showed that and had the same values. This led to the determination of the two lattice constants, *c* and α. The computer-generated XRD pattern revealed a strong peak for the (100) plane, regardless of the chosen values of *c* and α. By comparing the peak corresponding to the (100) plane with the measured diffraction pattern, it was possible to estimate the value of *c*. The value of *c* was then further refined using the Le Bail profile fitting algorithm with FOX software (Figure S10c).[20] The lattice constants were found to be in good agreement with the information obtained from the transmission electron microscopy (TEM) images. The guest-accessible void space was estimated to be 64.5% using the Mercury software (CCDC. Version 2024. 1.0).

Gas adsorption measurements
CO_2 adsorption isotherms (195 K) and N_2 isotherms (77 K) were obtained using an automated volumetric adsorption apparatus (BELSORP MAX, Microtrac-BEL) connected to a cryostat system (ULVAC-Cryo). Approximately 40 mg of **1-Ru**/**1-Rh** was added to each sample cell. Before adsorption measurements, the cells were degassed at 353 K for 12 h using the degassing function of the analyzer. The change in pressure was monitored, and the amount of adsorbed gas was determined based on the pressure decrease at equilibrium.

Photoreduction of carbon dioxide (CO_2)
A typical photoreduction experiment involved suspending 25 μg of **1-Ru** or **1-Rh** crystals in a 2.0 mL NMP solution or mixed solvent solutions containing 50 mM BIH, 0.10 M TFE, and 0.1 mM [Ir(ppy)$_3$]. The mixture was sonicated for 15 min unless otherwise specified. Subsequently, CO_2 was purged into the reaction vessel for 15 min. The solution was irradiated under a 300 W Xe lamp equipped with a 400 nm long-pass filter (Edmund Industrial Optics) to generate light in the 400–750 nm range at 20 ºC. The reaction was carried out in a custom-made aluminum box with a cooling system (Figure S11). The amounts of CO and H_2 produced in the headspace of the cells were quantified using a Shimadzu GC-8A instrument equipped with a thermal conductivity detector (TCD) and packed column containing molecular sieve 13X-S (60/80 mesh size). Calibration curves were obtained by injecting the known amounts of H_2 and CO standards.

$^{13}CO_2$ labeling experiment
A 2.0 mL NMP solution containing 25 μg of suspended **1-Ru** crystals, 50 mM BIH, 0.10 M TFE, and 0.1 mM [Ir(ppy)$_3$] was purged with Ar for 15 min. Subsequently, the solution was bubbled with $^{13}CO_2$ for 5 min. $^{13}CO_2$ gas was generated via the acidification of $Ba^{13}CO_3$ with 2.0 M HCl (98 at% ^{13}C; Sigma–Aldrich). Following the labeling step, the solution was irradiated for 28 h under a 300 W Xe lamp equipped with a 400 nm long-pass filter (Edmund Industrial Optics) to generate light in the range of 400 ≤ λ ≤ 750 nm at 20 °C. The reaction was carried out in a custom-made aluminum box with a cooling system. The evolved CO was detected by gas chromatography–mass spectrometer (GCMS) (Shimadzu GCMS-QP2020) equipped with an Rt®-Msieve 5A column (30 m length, 0.53 mm inner diameter, and 50 µm film thickness) using He as the carrier gas. The initial column temperature was set to 40 °C.

Table S1. Crystallographic data for **CHO-M** (M = Ru, Rh)

Compound	**CHO-Ru**·2THF[21]	**CHO-Rh**·2THF
CCDC	2235241	2342433
Formula	$C_{48}H_{52}O_{16}Ru_2$	$C_{48}H_{52}O_{16}Rh_2$
T / K	102	102
Formula weight	1087.03	1090.71
Crystal system	Triclinic	Triclinic
Space group	$P\bar{1}$	$P\bar{1}$
a / Å	10.1030(3)	10.0163(8)
b / Å	11.3908(4)	11.2663(9)
c / Å	11.9634(5)	11.9681(12)
α / °	63.205(4)	63.184(9)
β / °	71.840(3)	71.991(8)
γ / °	71.697(3)	71.758(7)
V / Å^3	1143.02(8)	1122.0(2)
Z	1	1
crystal size / mm^3	0.6×0.05×0.05	0.12×0.11×0.02
D_{calc} / g·cm^{-3}	1.579	1.614
F_{000}	556.0	558.0
λ / Å	0.71073	0.71073
μ(Mo Kα) / cm^{-1}	7.34	8.10
data measured	17717	15763
data unique	4679	4082
R_{int}	0.0335	0.0618
no. of observations	5149	5663
no. of variables	298	298
R_1^{a}	0.0249	0.0442
wR_2^{b}	0.0635	0.0845
Goodness of Fit	1.067	0.992

^{a} $R_1 = R = \Sigma||F_o|-|F_c||/\Sigma|F_o|$. ^{b} $wR_2 = [\Sigma w(F_o^2-F_c^2)^2/\Sigma w(F_o^2)^2]^{1/2}$

Table S2. Relevant bond lengths (Å) around the metal centers on **CHO-M** (M = Ru, Rh)

Compound	M–M/Å	Averaged M–O_{eq}/Å	M–O_{ax}/Å
CHO-Ru	2.2689(3)	2.0636	2.3578(12)
CHO-Rh	2.3856(6)	2.0348	2.308(2)

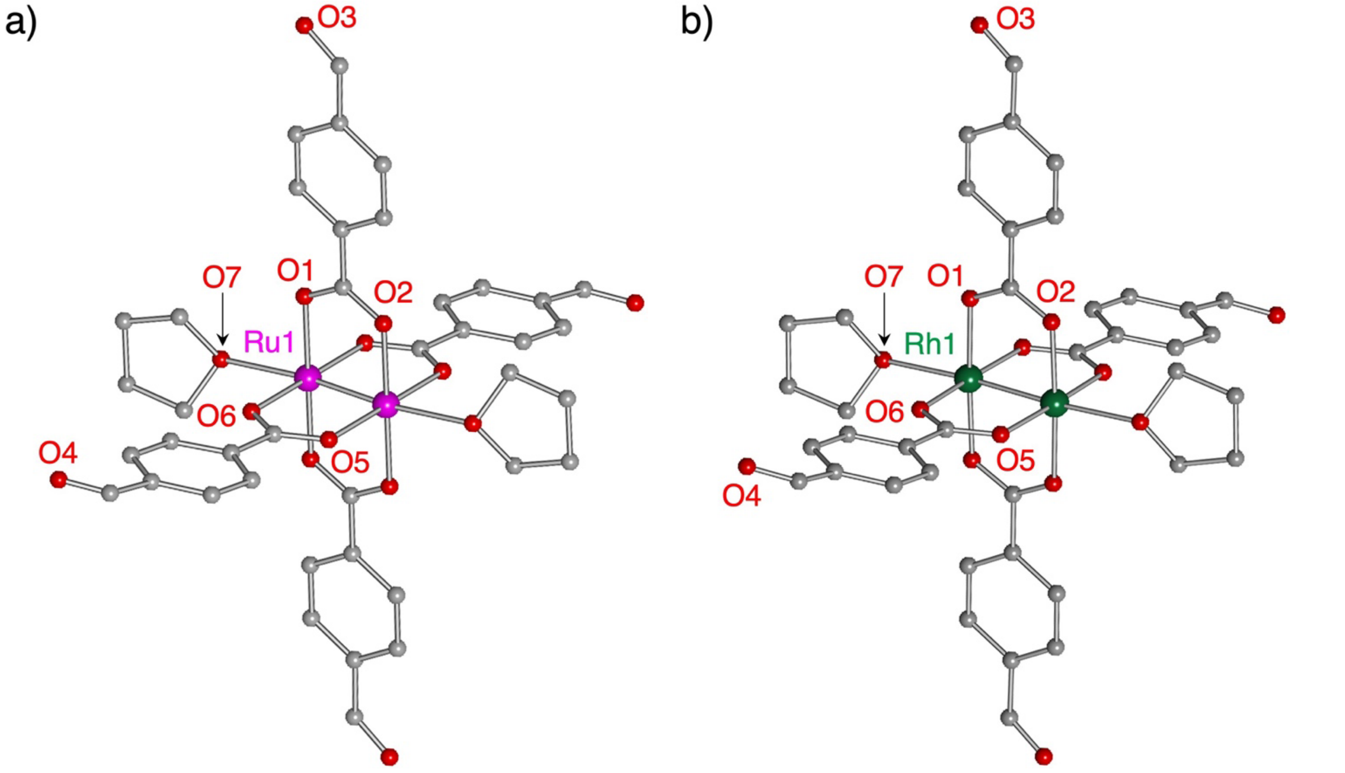

Figure S1. Structures of **CHO-Ru** (a) and **CHO-Rh** (b) (50 % probability ellipsoids), where the structure of **CHO-Ru** was referred from Ref. [21](CCDC-2235241).

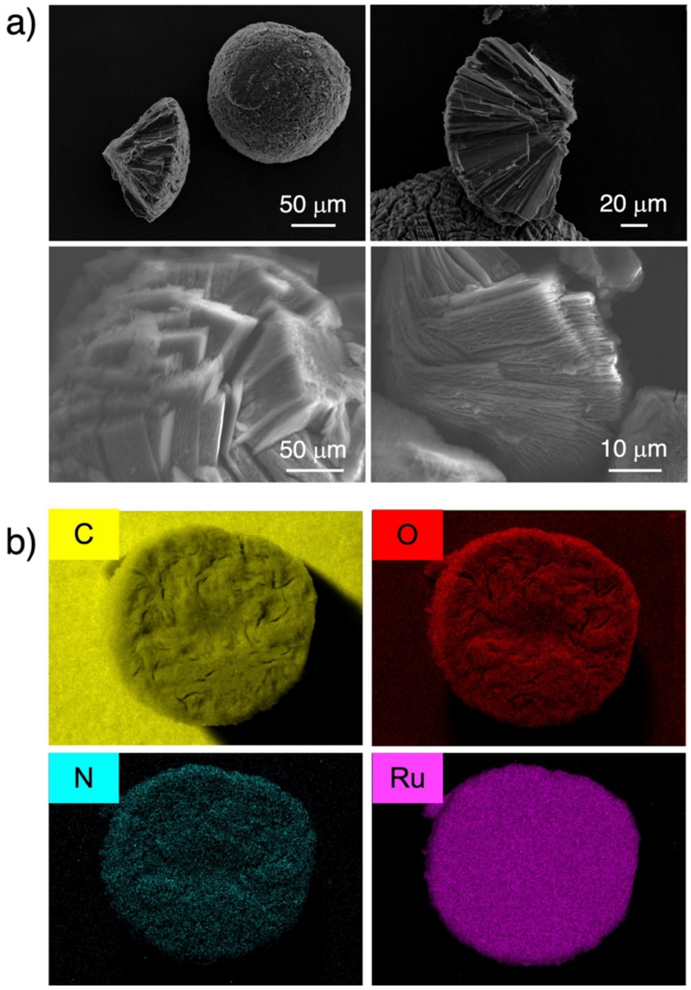

Figure S2. Additional SEM images (a) and element mapping images (b) of **1-Ru**.

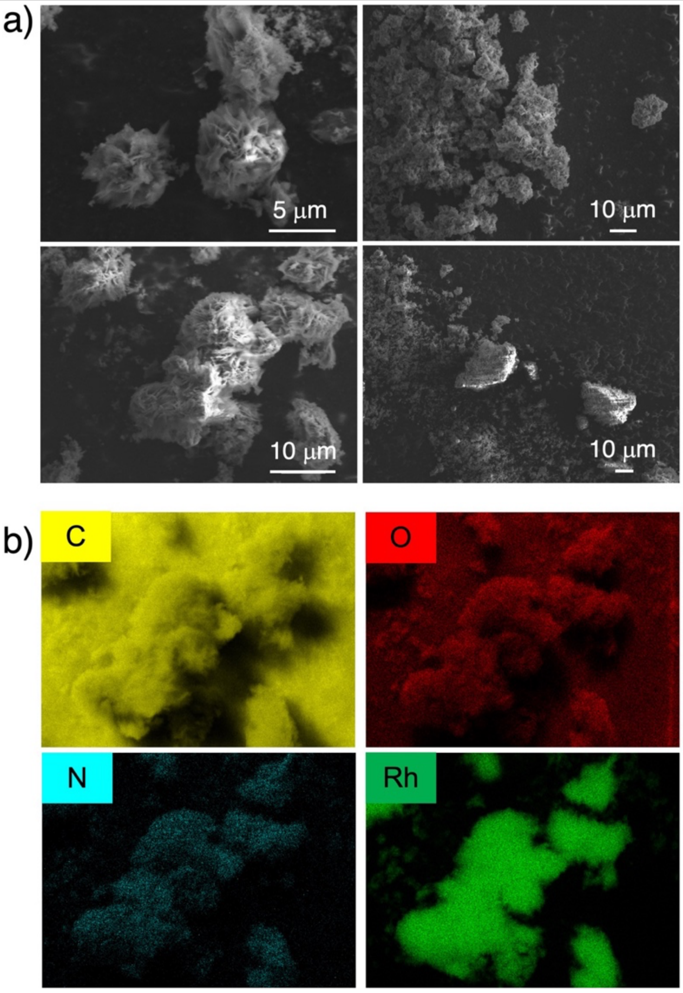

Figure S3. Additional SEM images (a) and element mapping images (b) of **1-Rh**.

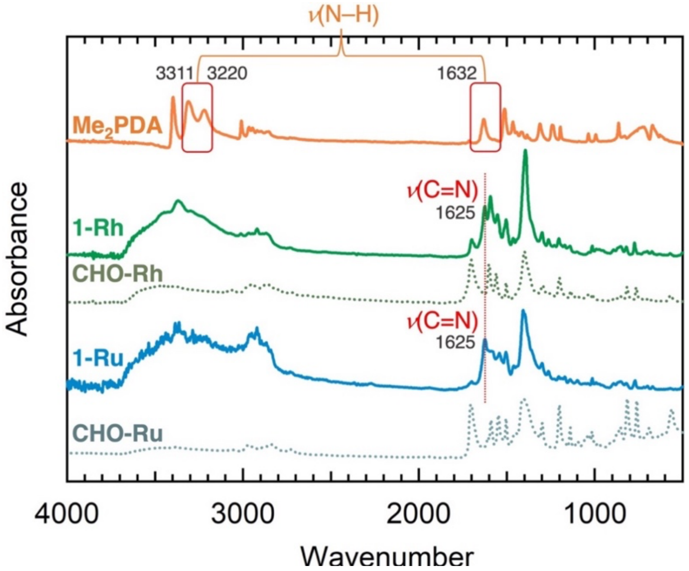

Figure S4. IR spectra in the range 500–4000 cm^{-1} for **1-Ru**, **1-Rh**, **CHO-Ru**, **CHO-Rh**, and Me_2PDA.

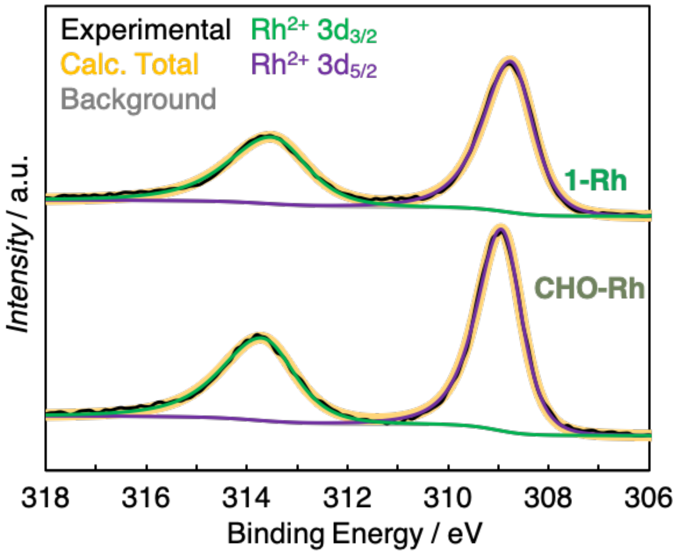

Figure S5. XPS for **CHO-Rh** and **1-Rh**. The spectra were assigned by Rh^{2+} 3d signals.

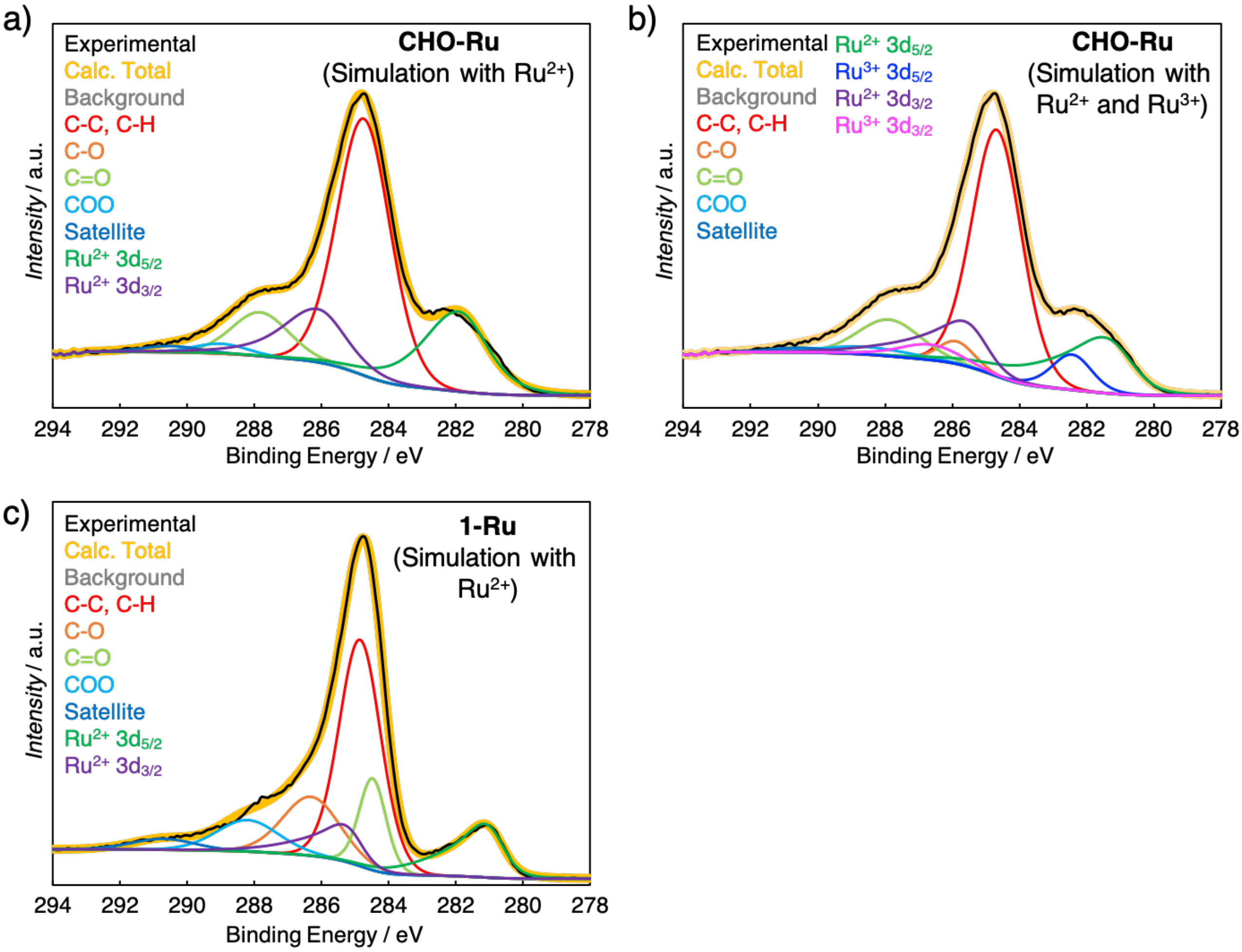

Figure S6. XPS for **CHO-Ru** (a, b) and **1-Ru** (c). Figure S6a for **CHO-Ru** was simulated with Ru^{2+} signals, whereas Figure S6b was simulated with Ru^{2+} and Ru^{3+} signals. Figure S6c for **1-Ru** was simulated with Ru^{2+} signals, whereas one with Ru^{2+} and Ru^{3+} was given in Figure 2b.

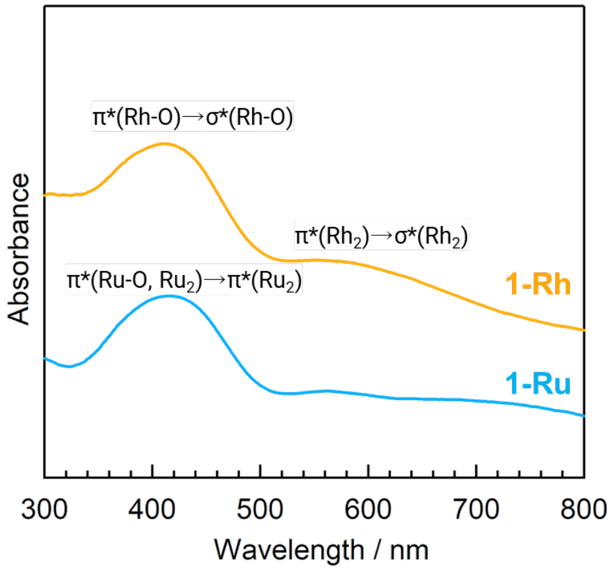

Figure S7. The solid-state UV-vis spectra of **1-Ru** and **1-Rh**. The absorbance bands at 411 nm, 549 nm for **1-Rh**, and 416 nm for **1-Ru** are assigned to π*(Rh–O)→σ*(Rh–O), π*(Rh_2)→σ*(Rh_2), and π*(Ru–O, Ru_2)→π*(Ru_2), respectively.[23, 24]

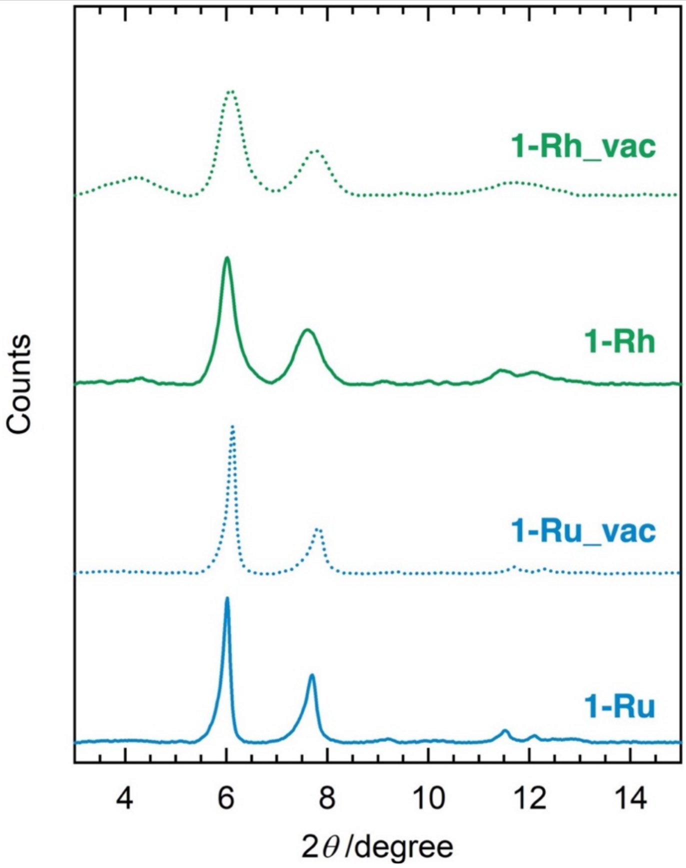

Figure S8. PXRD patterns of solvent-free dried samples (**1-M_vac**; M = Ru, Rh) of **1-Ru** and **1-Rh**, where **1-Ru** and **1-Rh** correspond to the as-synthesized samples.

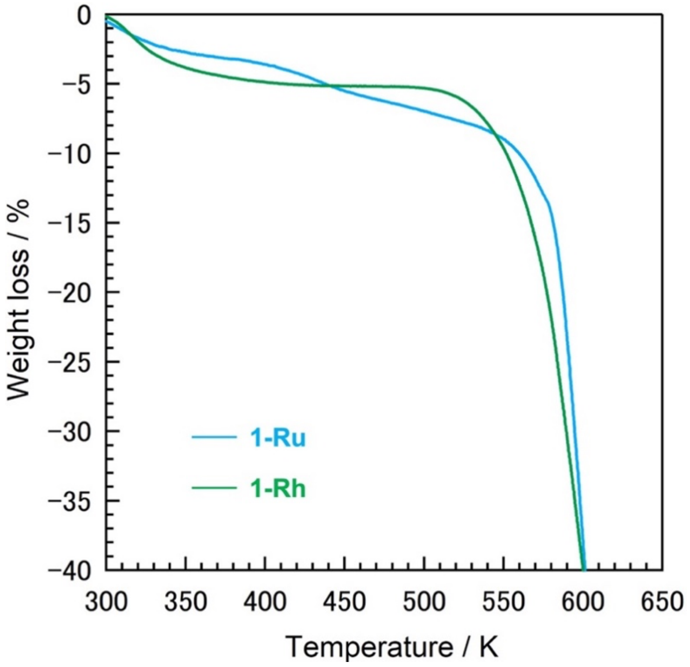

Figure S9. TGA profiles of as-synthesized samples of **1-Ru** and **1-Rh** with a heating rate of 5 K min^{-1}, where the vertical axis represents the relative weight loss from the measured sample.

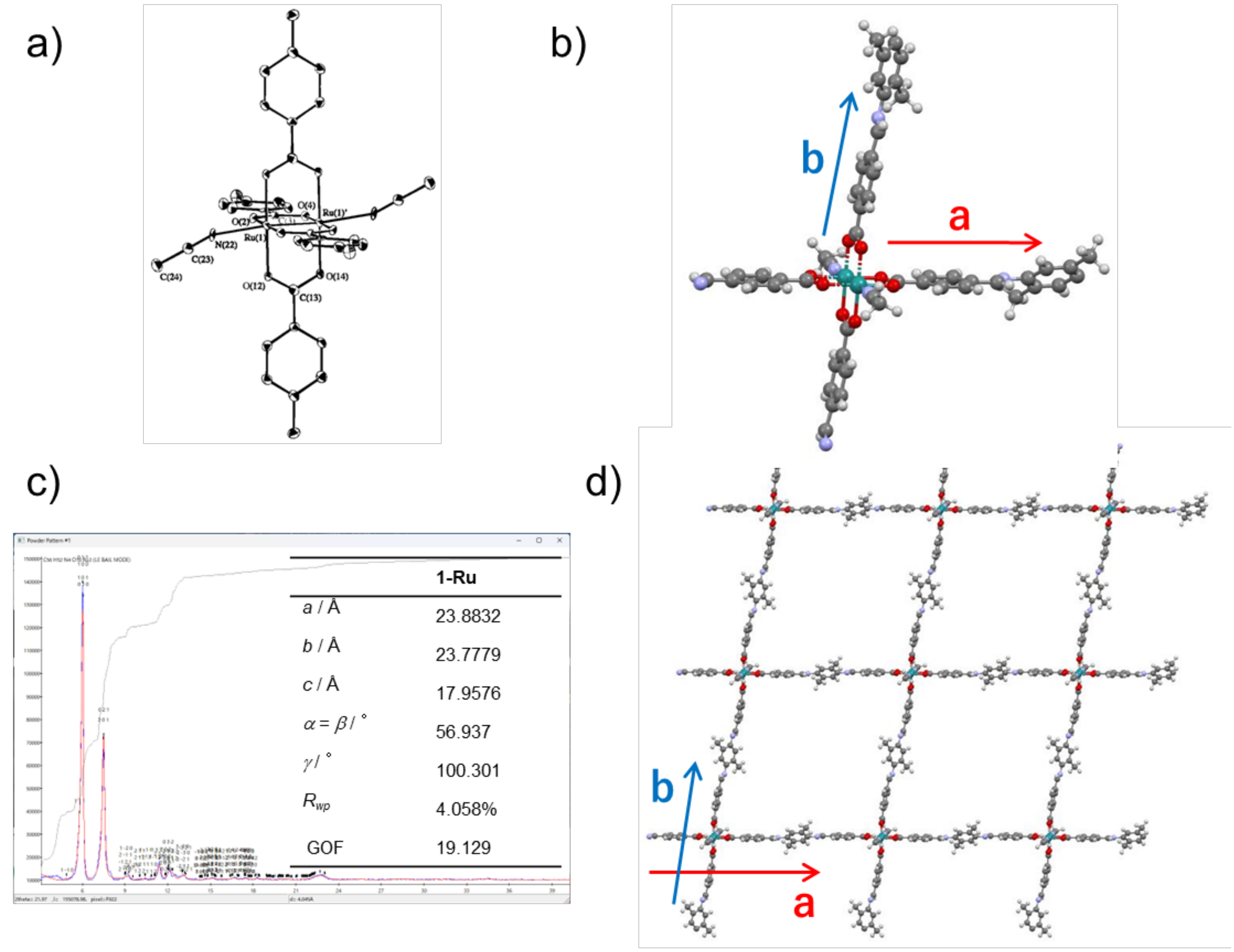

Figure S10. Structural model simulation of **1-Ru.** (a) Template crystal structure of **Me-[Ru$_2$]** referred from Ref.[22]. (b) Molecular model of the constituent units of **1-Ru**. (c) Le Bail analysis of **1-Ru**. Blue and red lines represent the observed plots and calculated pattern, respectively. (d) Two-dimensional planar structure of **1-Ru**.

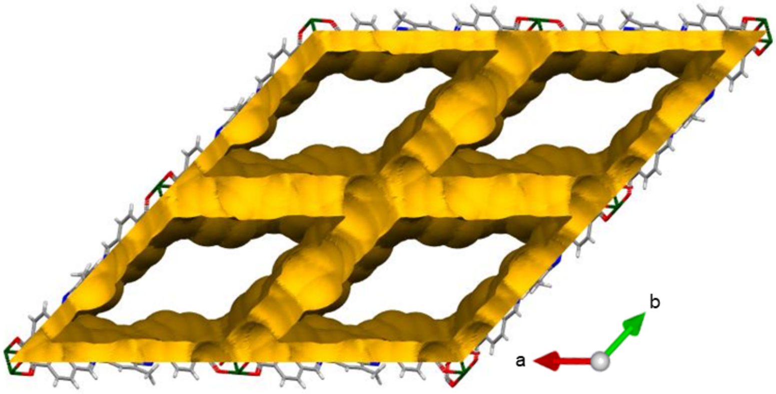

Figure S11. The pore volume of the model structure of **1-Ru**. The guest-accessible void space was estimated at 64.5% by using mercury software (CCDC. Version 2024. 1.0).

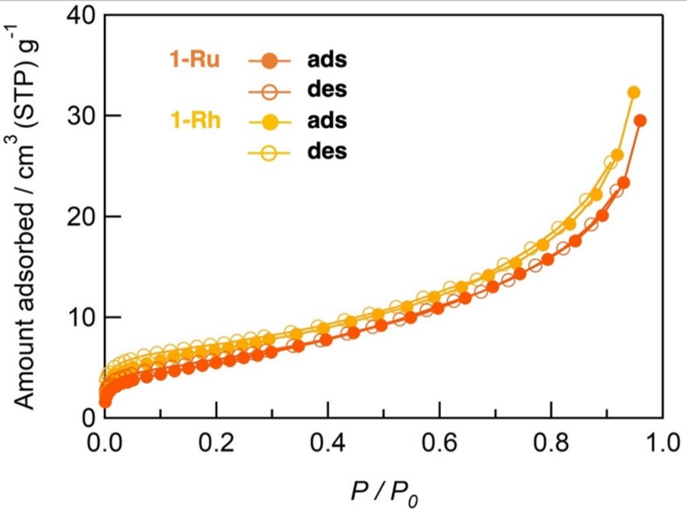

Figure S12. Adsorption isotherms of for **1-Ru** and **1-Rh** for N_2 at 77 K.

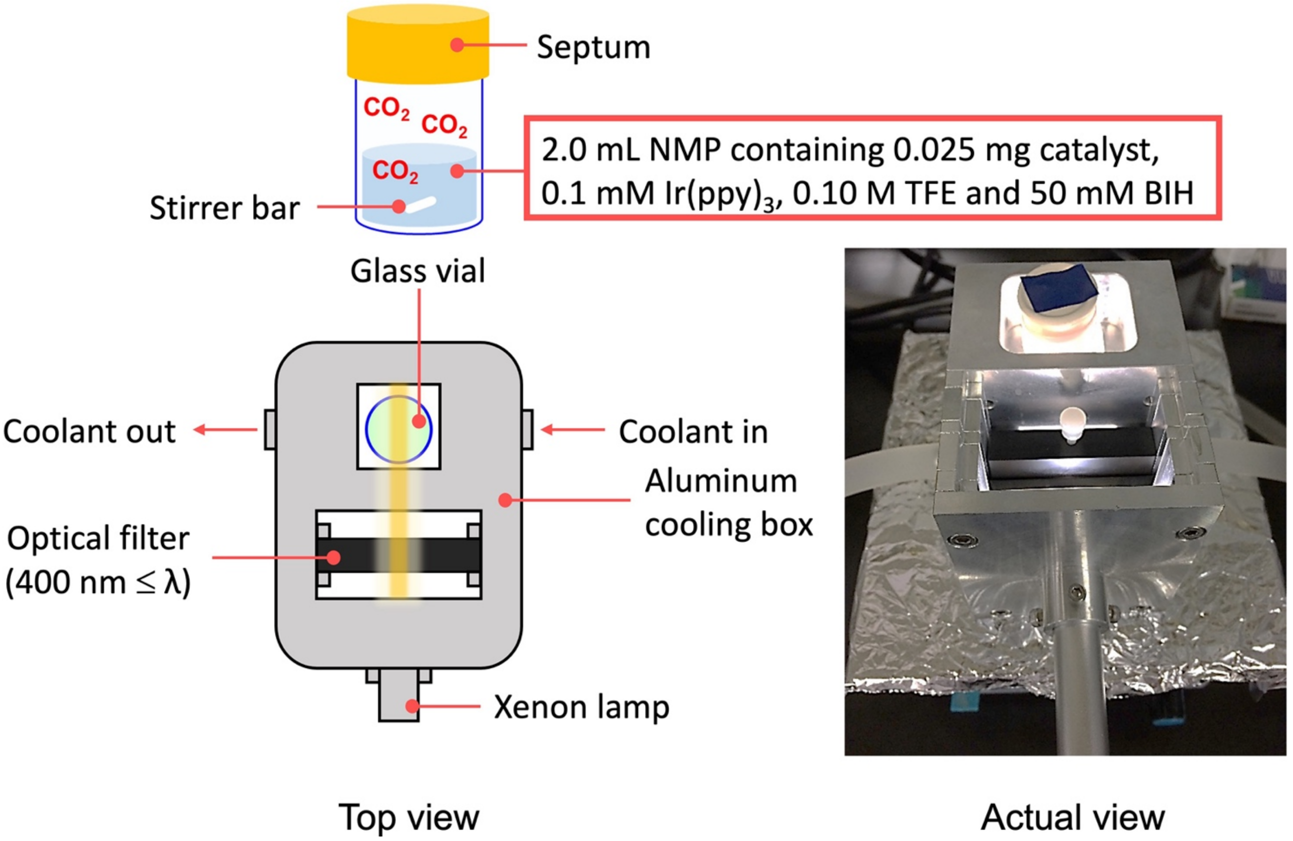

Figure S13. Custom-made photoreactor. A schematic illustration (left) and a photograph (right) are shown. The coolant temperature was set to 20 °C to maintain a constant sample temperature during the measurement.

Table S3. Control experiments for photochemical CO_2 reduction using **1-Ru** for 8 h.

Entry	catalyst[a]	photo sensitizer[b]	electron donor[c]	Proton source[d]	gas	light (nm)	Products (μmol g^{-1})	
							CO	H_2
1	**1-Ru**	[Ir(ppy)$_3$]	BIH	TFE	CO_2	$400 \le \lambda \le 750$	1.2×10^4	5.6×10^2
2	**1-Rh**	[Ir(ppy)$_3$]	BIH	TFE	CO_2	$400 \le \lambda \le 750$	8.8×10^2	4.1×10^3
3	–	[Ir(ppy)$_3$]	BIH	TFE	CO_2	$400 \le \lambda \le 750$	0	0
4	**1-Ru**	–	BIH	TFE	CO_2	$400 \le \lambda \le 750$	4.8×10^2	0
5	**1-Ru**	[Ir(ppy)$_3$]	–	TFE	CO_2	$400 \le \lambda \le 750$	7.4×10^2	0
6	**1-Ru**	[Ir(ppy)$_3$]	BIH	–	CO_2	$400 \le \lambda \le 750$	4.4×10^3	3.6×10^3
7	**1-Ru**	[Ir(ppy)$_3$]	BIH	TFE	Ar	$400 \le \lambda \le 750$	4.9×10^2	1.2×10^4
8	**1-Ru**	[Ir(ppy)$_3$]	BIH	TFE	CO_2	dark	0	0

[a]0.025 μg of **1-Ru** or **1-Rh**. [b]0.1 mM of [Ir(ppy$_3$)]. [c]50 mM of BIH. [d]0.1mM of TFE.

Table S4. Control experiments for photochemical CO_2 reduction by **1-Rh** for 8 h.

Entry	Catalyst[a]	photo sensitizer[b]	electron donor[c]	Proton source[d]	gas	light (nm)	products (μmol g^{-1})	
							CO	H_2
1	**1-Rh**	[Ir(ppy)$_3$]	BIH	TFE	CO_2	$400 \leq \lambda \leq 750$	8.8×10^2	4.1×10^3
2	–	[Ir(ppy)$_3$]	BIH	TFE	CO_2	$400 \leq \lambda \leq 750$	0	0
3	**1-Rh**	–	BIH	TFE	CO_2	$400 \leq \lambda \leq 750$	0	2.1×10^3
4	**1-Rh**	[Ir(ppy)$_3$]	–	TFE	CO_2	$400 \leq \lambda \leq 750$	2.5×10^2	2.1×10^3
5	**1-Rh**	[Ir(ppy)$_3$]	BIH	–	CO_2	$400 \leq \lambda \leq 750$	7.4×10^2	4.1×10^3
6	**1-Rh**	[Ir(ppy)$_3$]	BIH	TFE	Ar	$400 \leq \lambda \leq 750$	0	3.4×10^4
7	**1-Rh**	[Ir(ppy)$_3$]	BIH	TFE	CO_2	dark	1.5×10^2	5.7×10^3

[a]0.025 μg of **1-Rh**. [b]0.1 mM of [Ir(ppy$_3$)]. [c]50 mM of BIH. [d]0.1mM of TFE.

Table S5. Photocatalytic CO_2 reduction by **1-Ru** and **1-Rh** for 24 h in the presence of proton source.

Entry	catalyst[a]	photosensitizer[b]	electron donor[c]	Proton source[d]	gas	light (nm)	products (μmol g^{-1})	
							CO	H_2
1	**1-Ru**	$[Ir(ppy)_3]$	BIH	TFE	CO_2	$400 \leq \lambda \leq 750$	3.0×10^4	1.1×10^3
2	**1-Rh**	$[Ir(ppy)_3]$	BIH	TFE	CO_2	$400 \leq \lambda \leq 750$	3.2×10^3	4.8×10^3
3	**1-Ru**	$[Ir(ppy)_3]$	BIH	H_2O	CO_2	$400 \leq \lambda \leq 750$	9.8×10^3	1.1×10^3
4	**1-Rh**	$[Ir(ppy)_3]$	BIH	H_2O	CO_2	$400 \leq \lambda \leq 750$	3.9×10^3	4.6×10^4
5	**1-Ru**	$[Ir(ppy)_3]$	BIH	–	CO_2	$400 \leq \lambda \leq 750$	5.0×10^3	3.4×10^3
6	**1-Rh**	$[Ir(ppy)_3]$	BIH	–	CO_2	$400 \leq \lambda \leq 750$	2.7×10^3	4.4×10^3

[a]0.025 μg of **1-Ru** or **1-Rh**. [b]0.1 mM of $[Ir(ppy_3)]$. [c]50 mM of BIH. [d]0.1 M of TFE or 0.1 ml H_2O.

Table S6. Comparison of the catalytic activity of **1-Ru** and MCOF-based photocatalytic systems.

Catalyst	Photosensitizers	Electron donor	Solvent	Light/nm	CO Selectivity (%)	CO (μmol g^{-1})	Production rate (μmol g^{-1} h^{-1})	Ref
**1-Ru**	[Ir(ppy)$_3$]	BIH	NMP/TFE	300 W Xe lamp 400 ≤ λ ≤ 750	96	3.0×10^4	1.2×10^3	This work
Co-2.3-DHTA-COF	[Ru(bpy)$_3$]Cl$_2$	TEOA	MeCN/H_2O	300 W Xe lamp 420 ≤ λ	96	7.2×10^4	1.8×10^4	[25]
OMHS-COF-Co	[Ru(bpy)$_3$]Cl$_2$	TIPA	MeCN/H_2O	300 W Xe lamp 420 ≤ λ	92	4.5×10^4	1.6×10^4	[26]
CoPor-DPP-COF	[Ru(bpy)$_3$]Cl$_2$	TIPA	MeCN/H_2O	LED lamp 420 ≤ λ	82	2.0×10^4	1.0×10^4	[27]
Re-bpy-sp^{2}c-COF	–	TEOA	MeCN	300 W Xe lamp 420 ≤ λ	81	1.8×10^4	1.0×10^3	[28]
Re-COF	–	TEOA	MeCN	225 W Xe lamp 420 ≤ λ	98	1.5×10^4 (≥20 h)	7.5×10^2	[29]
H-COF-Ni	[Ru(bpy)$_3$]Cl$_2$	TEOA	MeCN/H_2O	Xe lamp 420 ≤ λ	96	5.7×10^3	2.3×10^3	[30]
Ni-TpBpy	[Ru(bpy)$_3$]Cl$_2$	TEOA	MeCN/H_2O	300 W Xe lamp 420 ≤ λ	96	4.1×10^3	8.2×10^2	[31]
CdS@COF	–	TEOA	MeCN/H_2O	300 W Xe lamp 420 ≤ λ	72	4.1×10^3	5.1×10^2	[32]

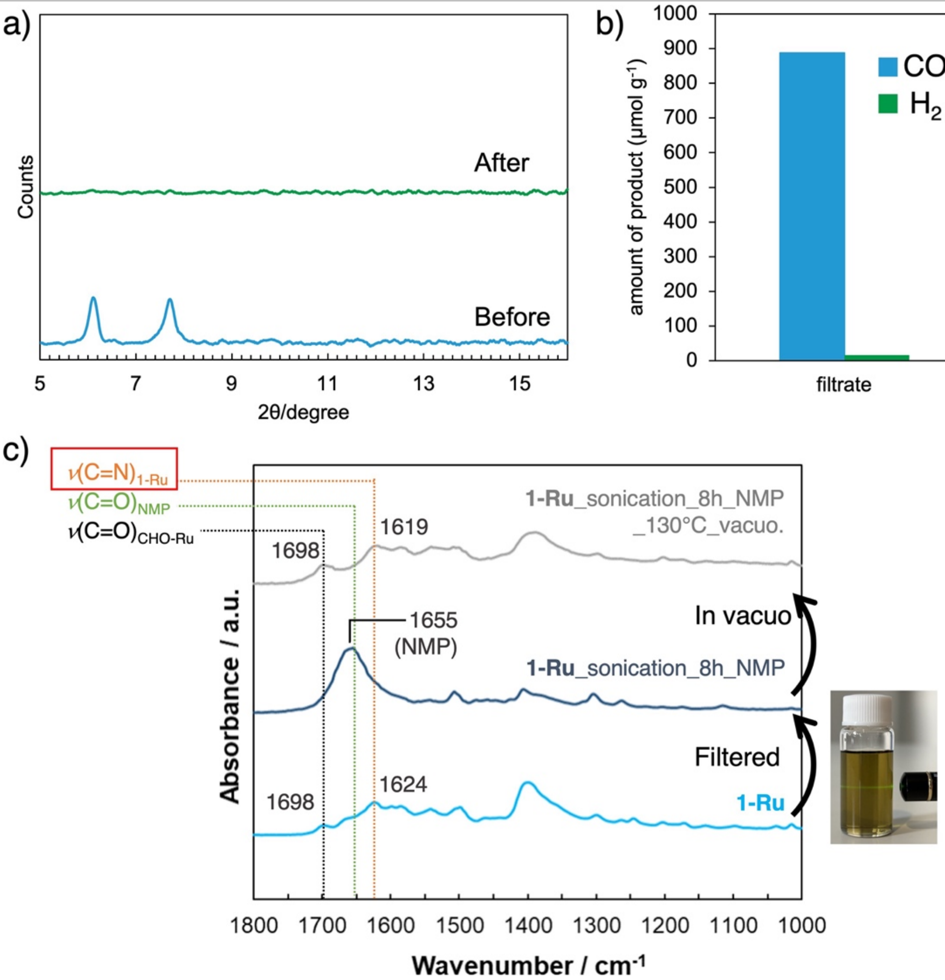

Figure S14. Investigation on recyclability of the **1-Ru** material. (a) PXRD patterns of pristine **1-Ru** and a residual powder obtained after filtering after one cycle reaction for 5 hours. (b) Catalytic activities of a system (in an NMP solution containing [Ir(ppy)3], TFE, and BIH at 20 ºC) using the filtered residual solid sample **1-Ru** (reaction time for 5 h). (c) FT-IR data on KBr pelletized samples for pristine sample, a residual sample after 8hours sonication (with NMP), and its dried sample obtained by evacuating at 130°C.

References

[1] R. W. Mitchell, A. Spencer, G. J. Wilkinson, *Chem. Soc., Dalton Trans.,* **1973**, 846.
[2] T. R. Felthouse, *Prog. Inorg. Chem.,* **1982**, *29*, 73–166.
[3] C. Itoh, H. Yoshino, T. Kitayama, W. Kosaka, H. Miyasaka, *Dalton Trans.,* **2024**, *53*, 444–448.
[4] E. A. Boudreaux and L. N. Mulay, *Theory and Applications of Molecular Paramagnetism*, John Wiley and Sons, New York, 1976.
[5] G. A. Bain, J. F. Berry, Diamagnetic Corrections and Pascal's Constants. *J. Chem. Educ.,* **2008**, *85*, 532−536.
[6] G. M. Sheldrick, SHELXT – Integrated Space-Group and Crystal-Structure Determination. *Acta Crystallogr., Sect. A: Found. Adv.,* **2015**, *A71*, 3−8.
[7] D. T. Cromer, J. T. Waber, in *International Tables for X-ray Crystallography Vol. 4* (Eds.: Ibers, J. A.; Hamilton, W. C.), The Kynoch Press: Birmingham, 1974, Table 2.2 A.
[8] J. A. Ibers, W. C. Hamilton, Dispersion corrections and crystal structure refinements, *Acta Crystallogr.*, **1964**, *17*, 781–782.
[9] D. C. Creagh, W. J. McAuley, in *International Tables for Crystallography Vol C* (Eds.: Wilson, A. J. C.), Kluwer Academic Publishers: Boston, 1992, pp. 219–222.
[10] D. C. Creagh, J. H. Hubbell, in *International Tables for Crystallography Vol C* (Eds.: Wilson, A. J. C.), Kluwer Academic Publishers: Boston, 1992, pp. 200–206.
[11] O. V. Dolomanov, L. J. Bourhis, R. J. Gildea, J. A. K. Howard, H. Puschmann, *OLEX2*: a complete structure solution, refinement and analysis program. *J. Appl. Cryst.,* **2009**, *42*, 339−341.
[12] M. H. Chisholm, G. Christou, K. Folting, J. C. Huffman, C. A. James, J. A. Samules, J. L. Wesemann, W. H. Woodruff, *Inorg. Chem.,* **1996**, *35*, 3643−3658.
[13] Gaussian 09, Revision E.01, M. J. Frisch, G. W. Trucks, H. B. Schlegel, G. E. Scuseria, M. A. Robb, J. R. Cheeseman, G. Scalmani, V. Barone, G. A. Petersson, H. Nakatsuji, X. Li, M. Caricato, A. Marenich, J. Bloino, B. G. Janesko, R. Gomperts, B. Mennucci, H. P. Hratchian, J. V. Ortiz, A. F. Izmaylov, J. L. Sonnenberg, D. Williams-Young, F. Ding, F. Lipparini, F. Egidi, J. Goings, B. Peng, A. Petrone, T. Henderson, D. Ranasinghe, V. G. Zakrzewski, J. Gao, N. Rega, G. Zheng, W. Liang, M. Hada, M. Ehara, K. Toyota, R. Fukuda, J. Hasegawa, M. Ishida, T. Nakajima, Y. Honda, O. Kitao, H. Nakai, T. Vreven, K. Throssell, J. A. Montgomery, Jr., J. E. Peralta, F. Ogliaro, M. Bearpark, J. J. Heyd, E. Brothers, K. N. Kudin, V. N. Staroverov, T. Keith, R. Kobayashi, J. Normand, K. Raghavachari, A. Rendell, J. C. Burant, S. S. Iyengar, J. Tomasi, M. Cossi, J. M. Millam, M. Klene, C. Adamo, R. Cammi, J. W. Ochterski, R. L. Martin, K. Morokuma, O. Farkas, J. B. Foresman, and D. J. Fox, Gaussian, Inc., Wallingford CT, **2016**.
[14] A. D. Becke, *J. Chem. Phys.,* **1993**, *98*, 5648−5652.
[15] D. Andrae, U. Häußermann, M. Dolg, H. Stoll, H. Preuß, *Theor. Chim. Acta.,* **1990**, *77*, 123−141.
[16] J. M. L. Martin, A. Sundermann, *J. Chem. Phys.,* **2001**, *114*, 3408−3420.
[17] R. Ditchfield, W. J. Hehre, J. A. Pople, *J. Chem. Phys.,* **1971**, *54*, 724−728.
[18] P. C. Hariharan, J. A. Pople, *Theor. Chim. Acta.,* **1973**, *28*, 213−222.
[19] W. J. Hehre, R. Ditchfield, J. A. Pople, *J. Chem. Phys.,* **1972**, *56*, 2257−2261.
[20] V. Favre-Nicolin, R. Černý, *J. Appl. Cryst.*, **2002**, *35*, 734-743.
[21] C. Itoh, H. Yoshino, T. Kitayama, W. Kosaka, H. Miyasaka, *Dalton Trans.,* **2024**, *53*, 444–448.
[22] M. H. Chisholm, G. Christou, K. Folting, J. C. Huffman, C. A. James, J. A. Samuels, J. L. Wesemann, W. H. Woodruff, *Inorg. Chem.,* **1996**, *35*, 3643–3658.
[23] V. M. Miskowski and H. B. Gray , *Inorg. Chem.*, **1988**, *27*, 2501.
[24] F. A. Cotton, E. A. Hillard, C. A. Murillo, *J. Am. Chem. Soc.*, **2002**, *124,* 5658.
[25] Q. Zhan, S. Gao, Y. Guo, H. Wang, J. Wei, X. Su, H. Zhang, Z. Liu, J. Wang, *Nat. Commun.*, **2023**, *14*, 1147.
[26] T. Zheng, X. Ding, T. Sun, X. Yang, X. Wang, X. Zhou, P. Zhang, B. Yu, Y. Wang, Q. Xu, L. Xu, D. Wang, J. Jiang, *Small.*, **2023**, 2307743.
[27] X. Wang, X. Ding, T. Wang, K. Wang, Y. Jin, Y. Han, P. Zhang, N. LI, H. Wang, J. Jiang, *ACS Appl. Mater. Interfaces.*, **2022**, *14*, 36 41122–41130.
[28] Z. Fu, X. Wang, A. M. Garfner, X. Wang, S, Y. Chong, G. Neri, A. J. Cowan, L. Liu, Z. Li, A. Vogel, R. Clowes, M. Bilton, L. Chen, R. S. Sprick, A. I. Cooper, *Chem. Sci.*, **2020**, *11*, 543–550.
[29] S. Yang. W. Hu, X. Zhang, P. He, B. Pattengale, C. Liu, M. Cendejas, I. Hermans, X. Zhang, J. Huang, *J. Am. Chem. Soc.*, **2018**, *140*, 14614–14618.
[30] S. Yang, R. Sa, H. Zhoung, H. Lv, D. Yuan, R. Wang, *Adv. Funct. Mater.*, **2022**, *32*, 2110694.
[31] W. Zhong, R. Sa, L. Li, Y. He, L. Li, J. Bi, Z. Zhuangm, Y. Yu, Z. Zou, *J. Am. Chem. Soc.*, **2019**, *141*, 7615–7621.
[32] L. Zou, R. Sam H. Zhong, H. Lv, X. Wang, R. Wang, *ACS Catal.*, **2022**, *12*, 3550–3557.

Author Contributions

H. M. designed the project. C. I. synthesized the present compounds and characterized them. H. Y. supported some of the experiments and measurements. M. Kitada., M. Kondo., and S. M investigated the photocatalytic activities. W. K., Y. O., and M. Kubo. conducted computational analysis and constructed the mode structure. J. M. and T. K. carried out the TEM measurements. H. M. mainly wrote the manuscript, and all authors finally edited it.
